# Chemical Dopants on Edge of Holey Graphene Accelerate Electrochemical Hydrogen Evolution Reaction

**DOI:** 10.1002/advs.201900119

**Published:** 2019-04-01

**Authors:** Akichika Kumatani, Chiho Miura, Hirotaka Kuramochi, Tatsuhiko Ohto, Mitsuru Wakisaka, Yuki Nagata, Hiroki Ida, Yasufumi Takahashi, Kailong Hu, Samuel Jeong, Jun‐ichi Fujita, Tomokazu Matsue, Yoshikazu Ito

**Affiliations:** ^1^ WPI Advanced Institute for Materials Research (AIMR) Tohoku University Sendai 980‐8577 Japan; ^2^ Graduate School of Environmental Studies Tohoku University Sendai 980‐856 Japan; ^3^ Institute of Applied Physics Graduate School of Pure and Applied Sciences University of Tsukuba Tsukuba 305‐8573 Japan; ^4^ Graduate School of Engineering Science Osaka University 1‐3 Machikaneyama Toyonaka 560‐8531 Japan; ^5^ Graduate School of Engineering Toyama Prefectural University 5180 Kurokawa Imizu Toyama 939‐0398 Japan; ^6^ PRESTO Japan Science and Technology Agency Saitama 332‐0012 Japan; ^7^ Max Planck Institute for Polymer Research Ackermannweg 10 55128 Mainz Germany; ^8^ Division of Electrical Engineering and Computer Science Kanazawa University Kanazawa 920‐1192 Japan

**Keywords:** chemical doping, electrochemical water splitting, graphene edge, noble metal‐free catalysts, scanning electrochemical cell microscopy

## Abstract

Carbon‐based metal‐free catalysts for the hydrogen evolution reaction (HER) are essential for the development of a sustainable hydrogen society. Identification of the active sites in heterogeneous catalysis is key for the rational design of low‐cost and efficient catalysts. Here, by fabricating holey graphene with chemically dopants, the atomic‐level mechanism for accelerating HER by chemical dopants is unveiled, through elemental mapping with atomistic characterizations, scanning electrochemical cell microscopy (SECCM), and density functional theory (DFT) calculations. It is found that the synergetic effects of two important factors—edge structure of graphene and nitrogen/phosphorous codoping—enhance HER activity. SECCM evidences that graphene edges with chemical dopants are electrochemically very active. Indeed, DFT calculation suggests that the pyridinic nitrogen atom could be the catalytically active sites. The HER activity is enhanced due to phosphorus dopants, because phosphorus dopants promote the charge accumulations on the catalytically active nitrogen atoms. These findings pave a path for engineering the edge structure of graphene in graphene‐based catalysts.

Metal‐free catalysts, in particular graphene‐based catalysts, have been utilized in fuel cells,^1^, ^2^, ^3^, ^4^ energy storage/conversion devices,^5^, ^6^, ^7^, ^8^, ^9^ as well as in the electrolysis of water,^10^, ^11^, ^12^, ^13^ due to their stable and controllable material properties. It is known that the catalytic activity on graphene‐based catalysts is largely affected by many factors such as conductivity,^14^, ^15^, ^16^ chemical dopants,^17^, ^18^, ^19^, ^20^ topological defects,^21^, ^22^ configuration of chemical bonding,^23^ chemical doping level,^24^ surface area,^24^ 3D‐porous structure,^25^, ^26^ and hybrid structures.^27^ Recent studies indicate that controlling these factors in a simultaneous manner can further enhance the catalytic activity;^14^, ^15^, ^16^, ^17^, ^18^, ^19^, ^20^, ^21^, ^22^, ^23^, ^24^, ^25^, ^26^, ^27^, ^28^ for example, Guo et al. revealed that chemical doping using pyridinic nitrogen atoms on a topologically controlled graphene edge enhances the oxygen reduction reaction in fuel cells.^23^ However, the reasons why controlling multiple factors can further enhance the electrochemical activity are still unclear. Direct observation and visualization of the catalytic activity at an atomic scale is a promising route to understand how multiple factors can have synergetic effects, allowing us to optimize the performance of carbon‐based metal‐free catalysts.

Electrochemical measurements have been used for visualizing catalytic activity at the nanoscale.^29^, ^30^, ^31^, ^32^ These measurements, combined with scanning tunneling microscopy,^29^ atomic force microscopy (AFM),^31^, ^33^ and optical instruments,^34^ can, in principles, probe the transient chemical species during electrochemical reactions, whereas practically these measurements are very difficult when the electrochemical current is applied. To overcome such difficulties, scanning electrochemical cell microscopy (SECCM) was developed very recently, which utilizes a nanopipette as a probe. This SECCM technique enables us to detect electrochemical current in sub‐microscale spatial resolution. Moreover, by using SECCM current mapping, one can analyze the electrochemical activities of the material with respect to the local structure. SECCM has been used to reveal the activities of the materials with the well‐defined structures, such as carbon nanotubes,^35^ graphene/graphite,^36^ polycrystalline Pt,^37^ and cleaved MoS_2_ nanosheets,^38^ as well as for Li‐ion redox reactions.^39^ Employing such a technique to understand the synergetic effect on the electrochemical activity is thus keen.

Here, by combining the SECCM technique with atomistic characterizations and density functional theory (DFT) calculations, we unveil the molecular mechanism behind the HER acceleration in edge‐enriched chemically doped graphene. We synthesized edge‐enriched graphene with chemical dopants near the edges created by holes in graphene lattices, and found that nitrogen/phosphorus (NP) codoped graphene provides much better HER activity than edge‐enriched graphene without any chemical dopants and edge‐enriched graphene with solo nitrogen (N) or phosphorous (P) dopants. The SECCM measurements reveal that the NP‐dopants on the edge of holey graphene significantly enhance the electrochemical current. The DFT calculation suggests that NP‐dopants situated on the edges enhance the contrast of the positively and negatively charges on atom's sites, lowering the Gibbs free energy of the HER process. A unique combination of the synthesis of the edge‐enriched doped graphene, SECCM measurement, and DFT calculation illustrates that chemical dopants located in the edges around the graphene edge structures improve the electrochemical properties, compared with edge‐free chemically doped graphene and edge‐enriched nondoped graphene. The obtained results provide insight into the rational design of metal‐free catalysts.

We synthesized N‐doped, P‐doped, NP‐codoped graphene with edge‐enriched structures by porous‐metal‐based chemical vapor deposition (CVD).^40^, ^41^ The upper panel of **Figure** **1**a displays schematics of the synthesis and expected edge‐enriched structures (Figure S1, Supporting Information), with the edges containing pyridinic‐type nitrogen (doped into edges), graphitic‐type nitrogen (doped into lattice), and tertiary phosphorous (doped into lattice) due to the intentionally prepared edge‐enriched graphene structures. The synthesized samples had bicontinuous and open porous morphologies, with ligament diameters of 0.4–1 µm and nearly identical pore sizes as each other (Figures S1 and S2, Supporting Information) tuned by catalytically inert SiO_2_ nanoparticle level.^41^ The edges along the hole with diameters ranged from 0.01 to 1 µm were observed by AFM (Figure S3, Supporting Information), and the average surface areas of edge‐enriched and edge‐free graphene samples, as determined by the nitrogen adsorption/desorption method with the Brunauer–Emmett–Teller (BET) theory,^42^ were 662.5 and 821.5 m^2^ g^−1^, respectively (Figure S4, Supporting Information). Raman spectra were measured in conjunction with the corresponding optical images to reveal the relationship between the edge‐enriched morphology and the graphene lattice. The defect density on the NP‐doped graphene with edge structures was indicated by the ratio of D and G bands in the Raman intensity spectra (*I*_D_/*I*_G_) (Figure 1b,c). The density of atom‐scale defects is highly localized near the edge, whereas the defect density is low in the region away from the edge. We also confirmed that the density of atom‐scale defects was high near the edges of 500–600 nm and 2 µm sized holes (see Figure S5 in the Supporting Information). Furthermore, the defect density in the edge region of the doped samples (*I*_D_/*I*_G_ = 0.80−0.91) were higher than that in the nondoped sample (*I*_D_/*I*_G_ = 0.72), implying that the dopants affected the defect density of the edge (Figure S6 and Table S1, Supporting Information). The number of graphene layers was indicated by the intensity ratio of the 2D and G bands (*I*_2D_/*I*_G_); the chemically doped graphene samples (*I*_2D_/*I*_G_ = 1.56−3.2) were composed mainly of several‐layer graphene, with high crystallinity of the graphene structures, and without detectable amorphous carbon features (Figure S7 and Table S1, Supporting Information). Therefore, we conclude that the curvature and ligament radii of the graphene are insensitive to the presence of edges; the edges only induce the high defect density in the edge regions due to their defective structures.

**Figure 1:**
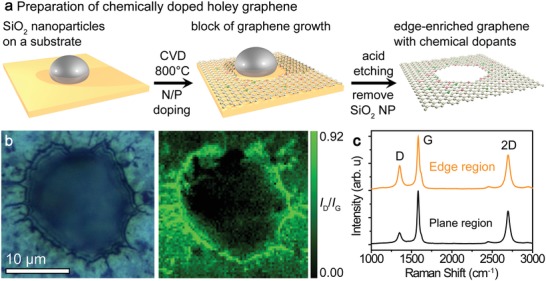
a) Schematic illustration of the preparation of edge‐enriched graphene with chemical dopants. b) Optical image and Raman mapping (*I*_D_/*I*_G_) of an edge region and c) the Raman spectra of NP‐doped graphene on the edge and plane regions.

Observations of atomic configurations and in situ elemental mapping were conducted with transmission and scanning transmission electron microscopy (TEM and STEM). The edge‐enriched chemically doped graphene demonstrates randomly oriented tubular ligaments composed of graphene layers (**Figure** **2**a). The inset of Figure 2a shows the sharp diffraction spots in the corresponding selected‐area electron diffraction pattern, evidencing that the sample has high crystallinity. High‐resolution (HR)‐TEM demonstrated that the majority of the plane region has a perfect hexagonal structure, as in 2D flat graphene (Figure 2b). In contrast, a defect‐induced distorted graphene lattice was observed in the edge region (Figure 2c). Moreover, bright‐field (BF)‐STEM images and elemental mapping of electron energy loss spectroscopy (EELS) data (Figure 2d) showed that the contrast in the edge region was relatively brighter than that far from the edges whereas the wrinkle edge region does not show (Figure S8, Supporting Information). This indicates that the chemical dopants were situated in the edge region, which is consistent with the high *I*_D_/*I*_G_ values found from Raman mapping (Figure 1b). Our results indicate that the chemical dopants are inhomogenously distributed and thus strongly illustrate that the edge region is the major host area of the dopants.

**Figure 2:**
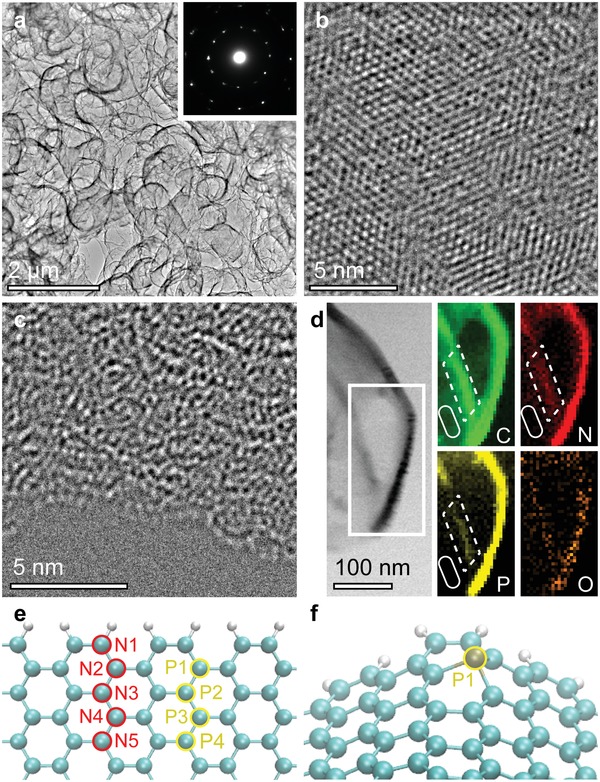
Atomistic characterizations of NP‐doped graphene with edge structures. a) Low‐resolution TEM image of the edge‐enriched graphene. The inset shows randomly orientated selected‐area diffraction spots of graphene layers. b) HR‐TEM image of the graphene on a plane region. c) HR‐TEM image of the graphene on an edge region. d) BF‐STEM image and corresponding EELS elemental mappings (C: carbon, N: nitrogen, P: phosphorus, O: oxygen) around an edge of NP‐doped graphene. Dot and solid lines represent edge and plane regions, respectively. e) Nondistorted graphene edge model for calculation of total energy (eV). f) Distorted edge model with a single phosphorus atom at P1 position.

The chemical binding state and quantitative chemical composition were investigated by X‐ray photoelectron spectroscopy (XPS) (Figures S9–S11, Supporting Information). The peaks in the C 1s XPS spectrum of the NP‐doped sample signify high quality graphene without any carbides. Furthermore, the N 1s XPS spectrum reveals that the N‐dopant forms graphitic, pyridinic, and oxide structures, without any nitrides;^43^, ^44^ and the P 2p XPS spectrum indicates that the binding state of phosphorus is that of tertiary phosphorous (P–C_3_) (132.1–132.9 eV).^45^, ^46^ The residue metal was analyzed and very tiny amounts of Ni and Mo impurities (≤0.01 at%) were observed on the surface (Figure S10 and S11, Supporting Information) by XPS measurements. Note that they were quantitatively estimated as less than ≈1 µg mg^−1^ by inductivity coupled plasma optical emission spectrometer analysis, while they could not be detected by X‐ray diffraction (XRD) (Figure S12, Supporting Information). The quantitative XPS measurements are summarized in Table S2 (Supporting Information). To understand the influence of the edges on the chemical binding, we compared the spectra of NP‐doped graphene with and without edges, grown under the same CVD conditions (Figures S9 and S13, Supporting Information). The existence of edges affected the total dopant level (1.03 at% increase), and the fraction of pyridinic nitrogen showed relatively higher atomic concentrations in the edge‐enriched NP‐doped sample (Table S2, Supporting Information).

To confirm the selective chemical doping into the graphene edge, we have performed DFT calculations with nondistorted and distorted (≈6% lattice shrinkage) graphene models to determine total energy of chemically doped graphene at the edge region (Figure 2e,f). The total energy was normalized at the position having the lowest total energy (i.e., N1 and P1) for comparison. The calculation results indicate that the total energy at the position of N1 and P1 is most energetically stable in comparison to other positions (0.19–0.59 eV for N and 0.40–0.67 eV for P) and the total energy increase as the chemical dopants are positioned away from the edge (**Table** **1**). Moreover, it was found that nitrogen dopants are doped into the distorted edge more preferable than those into the nondistorted edge (i.e., 0.1 eV reduction at each position at least). Furthermore, the phosphorus atom forms the pyramid‐like bonding with carbons (Figure 2f), which could stabilize the curved graphene edge lattice as observed (Figure 2a). Therefore, the chemical dopants could be predominately doped near the graphene edge. Combined with experimental results such as Raman mapping, high‐resolution (HR)‐TEM, EELS mapping, XPS data, and DFT calculations, we concluded that the edge can host chemical dopants and lead to the preferential configurations of dopants (especially pyridinic nitrogen in the edge). As a result, the mathematically suggested geometric frustration^47^ induced by edges plays an important role in accommodating more dopants and tailoring atomic configurations of pyridinic nitrogen, as has been reported previously.^21^ Such edge‐induced structural frustration is expected to affect the catalytic activity.

**Table 1:** Total energy (eV) of graphene edge lattice with a single chemical dopant relative to the N1 and P1 dopant positions. The lower value means that the dopant position is preferable

	Nondistorted/distorted edge				
	1	2	3	4	5
N‐doped graphene	0/0	0.32/0.19	0.59/0.48	0.52/0.41	0.43/0.30
P‐doped graphene	0/0	0.58/0.50	0.49/0.67	0.49/0.40	–

We subsequently measured the HER performance of solo N‐doped, solo P‐doped, and NP‐codoped graphene samples with edge‐enriched structures, as well as of nondoped (G) and edge‐free NP‐doped samples, together with the commercial Pt catalyst. **Figure** **3**a displays hydrodynamic voltammograms of the electrochemical HER at graphene cathodes in an acidic 0.5 m H_2_SO_4_ electrolyte. The overpotentials, η, at a current density of 10 mA cm^−2^, were 344 mV (NP), 477 mV (N), 522 mV (P), 571 mV (G), and 453 mV (edge‐free NP). Moreover, the approximate turnover frequencies (TOFs)^48^, ^49^ at electrode potentials of −200 mV (vs reversible hydrogen electrode, RHE) (Table S3, Supporting Information) were determined. NP sample (0.64 H_2_/s) exhibited a higher TOF than the edge‐free NP sample (0.45 H_2_ s^−1^). The NP‐sample exhibits the highest HER activities among the synthesized graphene samples. Electrochemical impedance spectroscopy was employed to investigate the charge transfer properties (Figure S14, Supporting Information). By assuming an equivalent circuit consisting of a parallel combination with a charge‐transfer resistances (*R*_ct_) and an electrochemical double‐layer capacitance (*C*_dl_), we can fit the half‐circle Nyquist plots to analyze their *R*_ct_ at an electrode potential of −300 mV (V vs RHE). The *R*_ct_ values were 21.5 Ω (NP), 73.2 Ω (N), 125 Ω (P), 1017 Ω (G), and 48.1 Ω (edge‐free NP). The NP‐sample clearly exhibits the lowest charge‐transfer resistance, which could accelerate the HER kinetics and increase catalytic activity. Both η and *R*_ct_ are lowered by the generation of edges on the NP‐sample, manifesting that the edges will enhance HER activities. The electrochemical stability over cycling and durability test at the potential of −0.34 V (V vs RHE) of the NP‐sample was examined in 0.5 m aqueous H_2_SO_4_ electrolyte (Figure S15, Supporting Information). The sample maintained 85% and 91.6% of its current density after 1000 cycles and 1 day, respectively.

**Figure 3:**
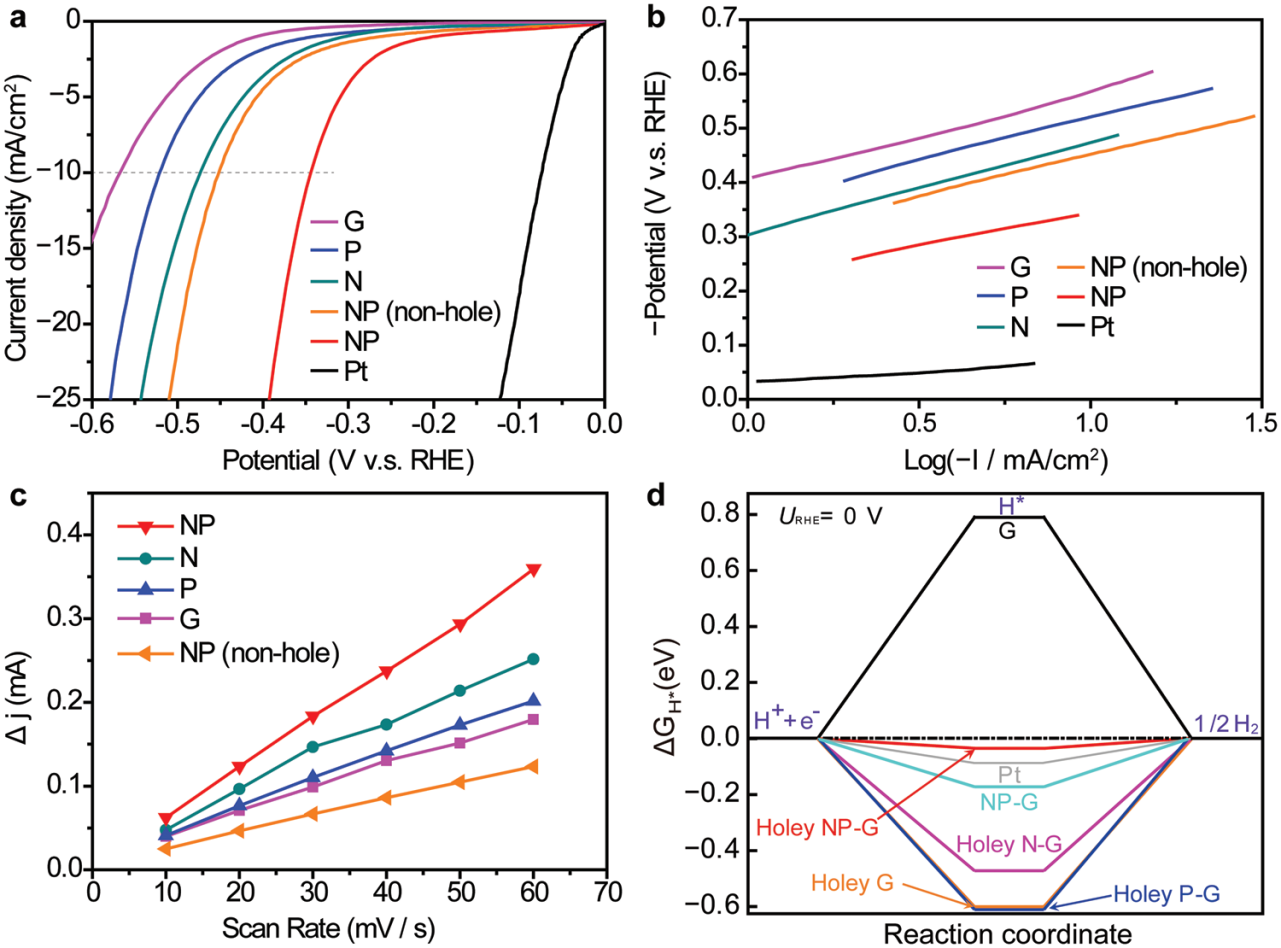
HER activities of chemically doped graphene with edge structures. a) Hydrodynamic voltammograms of graphene samples with and without edges in 0.5 m aqueous H_2_SO_4_ electrolyte. b) Tafel plots of the various graphene samples. c) Differences in current (Δ*J* =*J*_a_ − *J*_c_) at 150 mV (V vs RHE) as functions of scan rate. d) Gibbs free energy profiles calculated by DFT.

To obtain information about the catalytic activity, Tafel plots (Figure 3b) and *C*_dl_ (Figure 3c) of the graphene samples were examined. The Tafel slopes were of 118−171 mV dec^−1^, which suggests that the HERs occur via mixed Volmer and Heyrovsky mechanisms, i.e., the electrochemical desorption of adsorbed hydrogen (H_ads_) to generate hydrogen molecules determines the HER rate.^50^ The electrochemical surface area (ECSA), which is almost proportional to *C*_dl_, can be qualitatively compared by evaluating the *C*_dl_ values of the graphene samples on the basis of cyclic voltammetry (CV) at various sweep rates (Figure 3c and Figure S16, Supporting Information). The measured *C*_dl_ values were 14.1 mF cm^−2^ (NP), 10.4 mF cm^−2^ (N), 8.5 mF cm^−2^ (P), 7.4 mF cm^−2^ (G), and 5.2 mF cm^−2^ (edge‐free NP). The hydrophilic character was significantly enhanced by the addition of chemical dopants containing edges as confirmed by contact angles (Figure S17, Supporting Information). The chemically doped graphene samples with edge structures had decreased Tafel slopes and increased *C*_dl_ values, thus promoting HER. Moreover, we examined the dependence of edge/plane area ratio on hydrodynamic voltammograms to investigate the area balance, by preparing samples with various amounts of SiO_2_ nanoparticles which disturb graphene growth (1 × 10^−4^ to 1 × 10^−3^ wt%), as this correlates with edge and plane area by tuning the amount of edges (Figure S18, Supporting Information). Chemically doped graphene (no holes, SiO_2_: 0 wt%), less‐dosed edge graphene (10–30 nm holes, SiO_2_: 0.50 × 10^−4^ wt%), and overdosed hash graphene (100–300 nm holes, SiO_2_: 5.0 × 10^−4^ wt%) all have lower HER performances than that with an optimal amount of SiO_2_ nanoparticles (10–100 nm holes, SiO_2_: 1.0 × 10^−4^ wt%) under similar CVD conditions (Figure S19, Supporting Information). Furthermore, concentration dependences of chemical doping level were compared to examine the influence of chemical dopant level and holes (Figure S20, Supporting Information). The NP‐doped graphene (1.37, 2.42, and 3.45 at%) without holes demonstrates that higher chemical doping level shows higher HER performances which is consistent with previous reports.^4^, ^21^ Importantly, the NP‐doped graphene with holes (2.40 at%) demonstrates ≈10% lower overpotential at 20 mAcm^−2^ than NP‐doped graphene (2.42 at%) without holes. These indicates that creating an appropriate edge/plane area ratio at the same chemical doping level is important for enhancing the HER.

To link the local configurations with the electrochemical HER activity at a nanometer scale, we used operando electrochemical measurements for real‐space electrochemical mapping with SECCM. **Figure** **4** shows the topology and on‐site HER current for the NP‐doped and nondoped (G) graphene samples with the edge structures. The comparison between the topology data (Figure 4a) and the HER current mapping (Figure 4b) for the G‐sample demonstrates that the edge region has a larger HER current than the plane region. Furthermore, the HER current at the edge is enhanced by solo doping (Figure S21, Supporting Information) in comparison to the nondoping (Figures 4b) and further enhanced by codoping (Figures 4d) in comparison to the solo doping (Figures 4c,d). To compare the catalytic ability of the two regions, we estimated the TOF of hydrogen molecules from the HER current mapping data (Figure 4b,d) and summarized them in Table S3 (Supporting Information). We found that the TOF value of chemically doped graphene at the edge reach 468 H_2_ s^−1^ and the TOF values for chemically doped graphene were about 100–200 times higher at the edge than the plane regions, and about 10–80 times higher at the edge on the G‐sample than that for the plane regions on the G‐sample. This clearly indicates that the difference of HER current between the edge and plane regions is much larger for the NP‐sample than for the G‐sample. Thus, we can directly prove that combined chemical doping and edge‐engineering can enhance the HER activity drastically.

**Figure 4:**
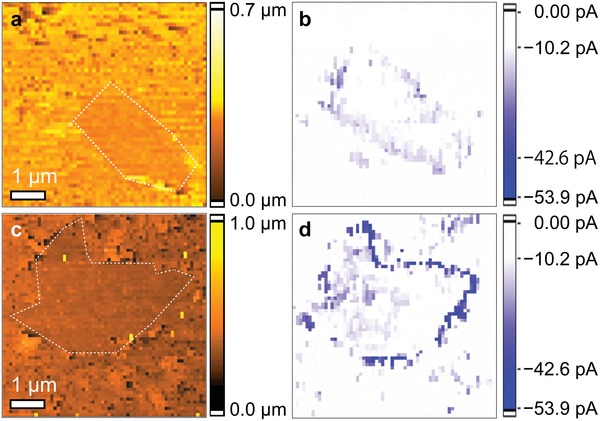
SECCM current mapping. a) Topography and b) HER current on nondoped graphene with edges and c) topography and d) HER current on NP‐doped graphene with edges. The current mapping obtained was −1000 mV vs Pd (equivalent to −200 mV vs RHE). The white dots in the topography images present the guidance of edges.

After confirming that the chemical dopants on the edges are important for enhancing HER activity, we set out to address how HER activity is enhanced. We carried out DFT calculations of Gibbs free energy, followed by charge distribution analyses on the samples. The overall HER mechanism in an acidic electrolyte involves three stages: from H^+^ + e^−^ to 1/2 H_2_ via H* adsorption.^51^, ^52^ For these processes, highly efficient HER catalysts should have Gibbs free energies for H* adsorption, |Δ*G*_H*_|, close to zero. The SECCM mapping results displayed in Figure 4d clearly illustrate that the NP‐doped graphene with edges has larger HER current than the nondoped graphene with edges, indicating that the activation barrier for electron transport is much smaller for the NP‐doped graphene than for the nondoped graphene. This is consistent with the much smaller |Δ*G*_H*_| of the NP‐doped graphene with edges than nondoped graphene with edges. The DFT calculation indeed suggests that |Δ*G*_H*_| of the NP‐doped graphene with edges is comparable with the |Δ*G*_H*_| of 0.08 eV for Pt. Note that the |Δ*G*_H*_| is calculated by choosing the highly active sites near edges (Figures S22–S24, Supporting Information). This was followed by N‐doped graphene with edges (Δ*G*_H*_ = −0.46 eV); however, the other samples have much larger |Δ*G*_H*_| values. Such a trend is consistent with the HER activity data (Figure 3a), where the NP‐samples with and without edges and N‐doped samples with edges have the highest, second‐highest and third‐highest HER activity, respectively. This indicates that the presence of the edge structure on the chemically doped graphene is crucial to lower |Δ*G*_H*_|.

The lowering of |Δ*G*_H*_| on the NP‐doped graphene with edge structures can be typically observed at pyridinic nitrogen atoms at the edge (Figures S22–S24, Supporting Information). To understand the mechanism behind the lowering of |Δ*G*_H*_|, we analyzed the charge distribution. The partial charge distributions of the edge atoms on the NP‐doped graphene with and without the edges (Figure S25, Supporting Information) At the edge, the nitrogen and carbon atoms neighboring phosphorous are negatively charged, and phosphorus and carbon atoms neighbouring nitrogen are positively charged. P‐dopants at the edge of graphene enhance the charge on the pyridinic nitrogen, which improves the activity of the pyridinic nitrogen. The edge structure also enhances charge accumulation on the carbon atoms near chemical dopants; in average, the charge is enhanced by 30–50%. This leads to high wettability of the graphenes (Figure S17, Supporting Information). Thus, we propose that positively and negatively charged edge regions induced by chemical dopants and defects create additional ECSA‐containing catalytic sites on the edge‐enriched graphene, as reported previously.^53^ Furthermore, the edge‐induced carbon atoms also could be chemically active sites (Figure S23).

Catalytic activity in chemically doped graphene with edge structures arises from the inhomogeneous spatial distribution of chemical dopants and their configuration of chemical binding. The edge brings geometrical frustration to the graphene lattice, which accumulates chemical dopants and tailors HER active configurations near the graphene edge, while defect‐less graphene layers in relatively low‐dosed plane regions support electron transfer to the edge regions. Thus, these regions cooperatively work to enhance the reaction kinetics. The DFT‐calculated Gibbs free energy and charge population demonstrate that the graphene edge tunes the H* adsorption and desorption, and creates positively and negatively charged states, thereby benefitting the HER processes.

The operando SECCM analysis allowed us to link the electrochemical HER current with graphene structures for visualizing where the catalytically active sites are. Only the chemically codoped edges demonstrate high HER activities by the synergetic effect of nitrogen and phosphorus codopants at edge‐enriched region. Conversely, edge‐free regions, or regions in which the chemical dopant level is relatively low, do not show high catalytic activities. This indicates that both chemical dopants concentrated near the edges (Figure 2d) and edge‐induced configurations in nanometer scale such as pyridinic nitrogen (Figure S26, Supporting Information) can be effective catalytic reaction sites. Indeed, the chemical binding after the 1000 cycle HER test indicates that pyridinic N is actively involved in the HER reaction and then the 0.66 at% pyridinic N lost or turned to oxidized N. Thus, pyridinic N takes a responsibility of HER. Moreover, we found that, compared with nitrogen atom, phosphorus atom is not strongly oxidized after the HER test. This indicates that the phosphorus dopants support charge accumulation of pyridinic nitrogen in HER. Furthermore, the conductive and bicontinuous graphene network, along with the optimal edge structure, facilitates mass and electron transport to the active sites. Consequently, by tuning the chemical doping level and chemical dopants' configurations, the edges associated with the chemical dopants significantly enhance their HER activity.

By combining HER performance measurements, electrochemical current probes through SECCM, atomic conformation obtained by HR‐TEM, and DFT calculations, we studied the electrochemical activity of chemically doped graphene with edge structures. We found that the combination of chemical doping and edge engineering can increase the HER activity drastically. To understand the underlying mechanism, we directly visualized the electrochemical HER current by SECCM, which suggested that chemical dopant‐enriched edge structures can effectively enhance electrochemical hydrogen evolution. The DFT calculation indicates that the contrast of the charge intensities of atoms at an edge is enhanced due to the presence of the edge, improving the activities of the pyridinic nitrogen. As a result, Gibbs free energies for H* adsorption in NP‐doped graphene with edge structures are similar to that of Pt. Such edge engineering approaches provide a good understanding and new design principles to exploit intrinsic metal‐free catalysts. Moreover, graphene‐based electrocatalysts have large space to further improve their catalytic activity when we suitably combine several enhancement factors such as conductivity, chemical dopants, topological defects, configuration of chemical bonding, chemical doping level, surface area, edges, and 3D porous structure.

## Conflict of Interest

The authors declare no conflict of interest.

## Supporting information

Supplementary
