# Supplementary material for: Chemical Dopants on Edge of Holey Graphene Accelerate Electrochemical Hydrogen Evolution Reaction
Source: Adv Sci (Weinh). 2019 Apr 1;6(10):1900119. doi: 10.1002/advs.201900119 (PMC6524082; doi:10.1002/advs.201900119)
Supplement: Supplementary file 1 — Supplementary [file ADVS-6-1900119-s001.pdf]

Copyright WILEY-VCH Verlag GmbH & Co. KGaA, 69469 Weinheim, Germany, 2019.

ADVANCED SCIENCE
Open Access

# Supporting Information

for *Adv. Sci.,* DOI: 10.1002/advs.201900119

## Chemical Dopants on Edge of Holey Graphene Accelerate Electrochemical Hydrogen Evolution Reaction

*Akichika Kumatani,* Chiho Miura, Hirotaka Kuramochi, Tatsuhiko Ohto,* Mitsuru Wakisaka, Yuki Nagata, Hiroki Ida, Yasufumi Takahashi, Kailong Hu, Samuel Jeong, Jun-ichi Fujita, Tomokazu Matsue, and Yoshikazu Ito**

Copyright WILEY-VCH Verlag GmbH & Co. KGaA, 69469 Weinheim, Germany, 2016.

# Supporting Information

**Chemical dopants on edge of holey graphene accelerate electrochemical hydrogen evolution reaction**

*Akichika Kumatani*, Chiho Miura, Hirotaka Kuramochi, Tatsuhiko Ohto*, Mitsuru Wakisaka, Yuki Nagata, Hiroki Ida, Yasufumi Takahashi, Kailong Hu, Samuel Jeong, Jun-ichi Fujita, Tomokazu Matsue, Yoshikazu Ito**

1. Materials and Methods

1.1 Preparation of graphene template of $NiMoO_4$ nanofiber

$NiMoO_4$ nanofibers were fabricated by a standard hydrothermal synthesis. Stock solution was prepared with 1.7 mmol of $Na_2MoO_4$ $2H_2O$ (+99.0%, Wako) and 1.7 mmol of $NiCl_2$ $6H_2O$ (+98.0%, Wako) in 20 ml distilled water.[S1−S3] The stock solution was stirring for 10 min and transferred into a 50 ml capacity of Teflon coated stainless steel autoclave and kept at 150°C for 12 h in a box type oven. The autoclave was naturally cooled to room temperature. The resulting product was washed with distilled water several times and collected with a centrifuge to remove impurities. After the purifications, the product was stored in distilled water.

1.2 Preparation of holey graphene

$NiMoO_4$ nanofiber solution was mixed with 18−27 nm $SiO_2$ nanoparticles (ST-50, 48 wt.%, Nissan Chemical Industries). The typical weight ratio of the $NiMoO_4$ nanofibers and $SiO_2$ nanoparticles is $10^{-4}$ wt.% ($SiO_2$) for graphene template. After stirring very well, the mixtures were casted on a Cu sheet as substrates and dried completely. The Cu sheet was employed to try to grow graphene on the entire NiMo surface including the surface facing to the substrate. The dried mixtures loaded on a ceramic plate were inserted into the center of a quatz tube (φ30×φ27×1000 mm) furnace and annealed at 900°C for 20 min to grow porous NiMo with the surface containing the $SiO_2$ nanoparticles under a mixed atmosphere of $H_2$ (100 sccm) and Ar (200 sccm). Then, the temperature sequentially set at 800°C and graphene was grown at 800°C for 40 sec under a mixed atmosphere of $H_2$ (100 sccm), Ar (200 sccm), benzene (0.1 mbar, Ardrich, 99.8%, anhydrous, non-doping), pyridine (0.2 mbar, Ardrich, 99.8%, anhydrous, nitrogen doping) and/or triphenylphosphine (90°C heating by an external heating device, Wako, 99%). The furnace was immediately opened and the inner quartz tube was cooled with a fan to room temperature. The samples were removed from the Cu sheets for measurements. For the non-holey graphene sample, the $SiO_2$ nanoparticles was not mixed with $NiMoO_4$ nanofiber solution and other procedures were similarly proceeded. The resulting graphene samples were dissolved with 1.0 M nitric acid for 2 days at 50°C. During the acid treatment, bubbles were generated on the interface between $SiO_2$ nanoparticles and NiMo surface, removing the $SiO_2$ nanoparticles from the surface. Then, the samples were further dissolved with 5% ammonia solution for 1 day at 50°C mainly for Mo dissolution. After dissolving Ni and Mo impurities, the graphene samples were thoroughly washed by ultrapure water 5 times and stored in ultrapure water.

1.3 Preparation of holey graphene for Raman mapping and SECCM experiments

$NiMoO_4$ nanofiber solution was mixed with 18−27 nm $SiO_2$ nanoparticles (ST-50, 48 wt.%, Nissan Chemical Industries). The weight ratio of the $NiMoO_4$ nanofibers and $SiO_2$ nanoparticles is $10^{-3}$ wt.% ($SiO_2$) for graphene templates. After stirring very well, the mixtures were casted on a Cu sheet as substrates and spin-coated to reduce roughness of samples as much as possible. The Cu sheet was similarly employed for graphene growth with same conditions as described in section 1.2. After the CVD process, the samples on the Cu sheets was dissolved with 1 M Iron(III) nitrate solution for 1 day and 1 M nitric acid solution for two days. After washing Cu, Ni and Mo impurities with ultra-pure water thoroughly, the graphene samples were transferred to $SiO_2$ glass substrate for Raman mapping and Nb-doped $SrTiO_3$ substrate for SECCM.

2. Microstructure characterization and property measurements

2.1 Imaging and spectroscopic characterization

The microstructure of chemically doped holey graphene was characterized by a scanning electron microscope (SEM, Hitachi S4800) and transmission electron microscope (JEOL

JEM-2100F and JEM-ARM200F) equipped EELS analysis (GATAN Enfinium). The samples were transferred on a Cu grid without a carbon support film.

2.2 Raman spectroscopy

Raman spectra were recorded by using a micro-Raman spectrometer (Renishaw InVia Reflex 532) with an incident wavelength of 532.5 nm. The laser power was set at 5.0 mW to avoid possible damage by laser irradiation. The holey graphene samples were placed on a background-free glass slide. The accumulation time of each spectrum is 400 s. The Raman mapping images were acquired by a confocal Raman spectroscopic system (Renishaw InVia Reflex) with 532.5 nm excitation laser. The intensity ratio of D band and G band mapping was obtained by the Gaussian-Lorentzian function on the equipped software of WiRE 4.3.

2.3 BET surface area and BHJ measurements

Nanopore sizes and surface areas of the holey graphene samples were measured by the Brunauer-Emmett-Teller (BET) methods using a BELSORP-MAX (BEL. JAPAN. INC) at 77.0 K. The horizontal axis was normalized with the vapor pressure of nitrogen ($P_0$) at 77.0 K (= 0.101 MPa). Samples were heated at 300°C under vacuum for 24 hours before the measurements.

2.4 XRD measurements

XRD was carried out using a PANalytical X'pert (Malvern Panalytical) with a rotating anode generator (Cu Kα1 radiation; λ = 1.5406 Å). The samples were loaded on a Si substrate.

2.5 XPS measurements

The chemical binding states of the chemically doped holey graphene were studied by X-ray photoelectron spectroscopy (XPS, AXIS ultra DLD, Shimazu) with Al *K*a and X-ray monochromator.

2.6 Electrochemical characterizations

Hydrodynamic voltammetry for HER and electrochemical impedance spectroscopy were conducted using an electrochemical workstation (Biologic, VSP-300) equipped with a rotation disk electrode (RED, 5.0 mm diameter glassy carbon, HOKUTO DENKO corp.). A graphite plate, an Ag/AgCl electrode (HOKUTO DENKO corp.) and the graphene samples (lording amount: 3.0 mg/cm$^2$) dispersed on the glassy carbon served as a counter electrode, the reference electrode and the working electrode, respectively. The Ag/AgCl electrodes was compared with a flesh Ag/AgCl electrode to check the differences of potential before use. However, all of the electrode potentials in the text were represented with respect to the corresponding reversible hydrogen electrode, i.e. by adding a value of (E(Ag/AgCl) + 0.059pH + 0.1976) V. The pH values (0.5) at room temperature were measured before HER tests. The CV measurements after several cycles were recorded from −850 mV to +100 mV (v.s. RHE) at a sweep rate of 10 mV/s in 0.5 M $H_2SO_4$ solution deaerated with Ar (99.999%) at room temperature with the disk rotation speed of 1600 rpm to remove generated hydrogen bubbles from the electrode surface. The obtained CV curves were calculated for an average of the current from the forward and the reverse sweeps to remove the capacitive background.[S4-S6] The electrode potential in the hydrodynamic voltammogram was automatically iR-compensated with the Ohmic resistance measured at +200 mV (v.s. RHE). Electrochemical impedance spectroscopy was carried out at −200 mV (v.s. RHE) with an amplitude of 50 mV. The durability of the electrodes was tested by potential cycling between −200 mV to +100 mV (v.s. RHE) at 10 mV/s. The double-layer capacitance, $C_{dl}$, were estimated on the basis of the cyclic voltammograms collected between 100 and 240 mV (vs. RHE) in 0.5 M $H_2SO_4$ solution at room temperature with various sweep rates from 10 to 60

mV/s. The details have been reported somewhere.[S7-S13] The ratio of TOF values of the holey chemically doped graphene catalysts were roughly calculated by the equation [S14]:

$$\mathrm{TOF} = j/nFN$$

Where $j$ is the current density at overpotential at 200 mV, $n$ is the stoichiometric number of electrons consumed in the electrode reaction (i.e., n = 2 for the HER), $F$ is the faraday constant and $N$ is the value of number of active sites under an assumption of equal number of chemically active site.

2.7 Contact angle
The contact angle of holey graphene and non-holey graphene were measured with standard contact angle measurements (VCA Optima-XE, AST products, Inc.). The samples were fixed on a double-stick tape and a 1 μl water droplet was manually doped on the surface of graphene samples.

2.8 Inductivity coupled plasma optical emission spectrometer (ICP-OES)
Grapheme samples were dissolved in aqua regia. The diluted solution was analyzed by inductivity coupled plasma optical emission spectrometer (ICP−OES, ICPS−8100, Shimazu) to determine the weight of Ni and Mo.

3. DFT calculations
We performed first-principle calculations with the VASP code[S15], using the projected augmented wave (PAW) [S16] method. For the exchange–correlation functional, we used the Perdew–Burke–Ernzerhof (PBE) [S17] functional. The plane wave energy cutoff was set to 400 Ry. The dispersion correction was included via Grimme's D3 method with Becke–Johnson (BJ) damping [S18-19]. The lattice constant of the graphene was taken as 17.04 × 19.676 Å, corresponding to the orthorhombic 4 × 8 cell of graphene. To avoid the artificial interactions between graphene layers under the periodic boundary condition, a 20 Å vacuum was inserted. The Brillouin zone was sampled with a Monkhorst-Pack 4 × 4 × 1 k-grid. The holey structures and positions of dopants (N and P) were constructed from the XPS results and EELS mapping (**Figure S22**). The size of the hole is approximately 1 nm. The pyridinic-type nitrogen atoms are doped into fringes, while the graphitic-type nitrogen atoms and tertiary phosphorous atoms are doped into the lattice.
We adsorbed hydrogen atoms and calculated the adsorption energy. The hydrogen adsorption energy ($\Delta E_H$) was calculated as

$$\Delta E_H = \frac{1}{n}\left(E_{tot} - E_{sub}\right) - \frac{1}{2}E_{H_2},$$

Where $E_{tot}$ is the total energy of the substrate with $n$ hydrogen atom adsorbed on the surface, $E_{sub}$ is the total energy of the substrate, and $E_{H2}$ is the energy of a hydrogen molecule in the gas phase (about −6.7 eV was employed in this work). The Gibbs free energy for the hydrogen absorption was corrected as

$$\Delta G_H = \Delta E_H + \Delta E_{ZPE} - T\Delta S_H,$$

where $\Delta E_{ZPE}$ is the difference in zero-point energy between the adsorbed hydrogen and hydrogen in the gas phase and $\Delta S_H$ is the entropy difference between the adsorbed state and the gas phase. As the contribution from the vibrational entropy of H in the adsorbed state is

negligibly small, the entropy of hydrogen adsorption is $\Delta S_H \approx -1/2S_H$, where $S_H$ is the entropy of $H_2$ in the gas phase. Then the Gibbs free energy with the overall corrections can be calculated as $\Delta G_H = \Delta E_H + 0.24$ eV.

To reveal the active sites at the atomistic level, we calculated the Gibbs free energy of individual atoms on the fringe (Figure S16). The $\Delta G_{H^*}$ values of carbon atoms are too negative, due to the strong binding energy between the hydrogen atom and the carbon atom on the fringe. The results for N-graphene clearly show that the pyridinic nitrogen is highly active compared with carbon atoms. The activities of pyridinic nitrogen atoms significantly improve with P-doping, indicating that the pyridinic nitrogen is the active site of NP-doped holey graphene. (**Figure S22-S24**). We also found that the $\Delta G_{H^*}$ of the pyridinic nitrogen (−0.12 eV to −0.26 eV) on the fringes improved in comparison to chemically doped graphene without holes (0.68 eV to 0.79 eV) [S20].

To calculate the $\Delta G_{H^*}$ values of the systems, three hydrogen atoms are adsorbed on atoms at the fringe as shown in Figure. S17. The active sites having low $\Delta G_{H^*}$ values were chosen as the hydrogen adsorption sites at the fringe, i.e. the pyridinic nitrogen atoms for N-graphene and NP-graphene, and carbon atoms for graphene and P-graphene. The $\Delta G_{H^*}$ value of NP-doped graphene without holes was taken from Ref. S15. The NP-doped holey graphene showed the lower value of $\Delta G_{H^*}$ (−0.02 eV), because of the extremely high activity of the pyridinic nitrogen. We also calculated $\Delta G_{H^*}$ values with the RPBE functional[S21], which slightly underestimates the adsorption energy on metal surfaces[S22], and obtained very similar results with PBE (Table S4). Finally, the density of states of C atoms indicate that the chemical dopants enhance the electronic densities of state at the Fermi level, which is related to electrical conductivity (**Figure S28**), resulting in the promotion of electrochemical hydrogen production [S23].

4. SECCM experimental conditions

Instrumental details of the SECCM system were described in past our report [S24]. The nanopipette was prepared by pulling a borosilicate glass capillary (GC150F-10 from Harvard Apparatus), which is applied as a probe of the SECCM. The diameter of nanopipette was typically 70 nm as shown in Figure S27. 0.5 M $H_2SO_4$ and a palladium (Pd) wire were filled as aqueous acid electrolyte and a quasi-reference counter electrode (QRCE). For QRCE, the Pd wire was used as a stable reference electrode under acid condition for HER measurement. As the pipette was in proximity to the sample, the electrolyte in the pipette created a meniscus on the sample surface, which behaved as a nanoscale electrochemical cell simulator. During approach process, the −1000 mV versus Pd QRCE (equivalent to −200 mV vs. RHE) was applied between the graphene sample and the PD wire. Once the meniscus was created, the capacitive current was mainly detected. At the instant the current was detected, the approach was stopped, recording as height information. Then, 3 milliseconds was waited for suppressing the capacitive current, introducing the current response related to HER for 50 microseconds. The HER active sites was visualized on the graphene sample by repeating the process to create the meniscus at each measurement point. Notably that the interval between each meniscus was separated over 70 nm to ensure that measurement points were not interfered each other. For more details of scanning protocols and programs were described in reference S24.

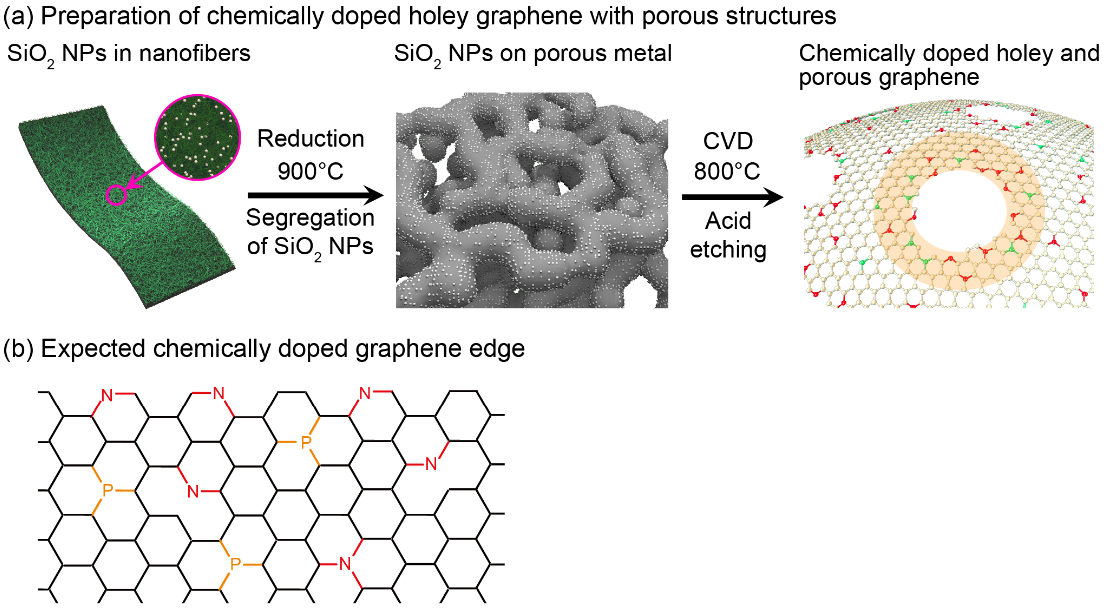


Figure S1. (a) Schematic illustration of the preparation of holey chemically doped porous graphene. The orange area defines the fringe region near a hole. (b) Expected chemically doped graphene edge.

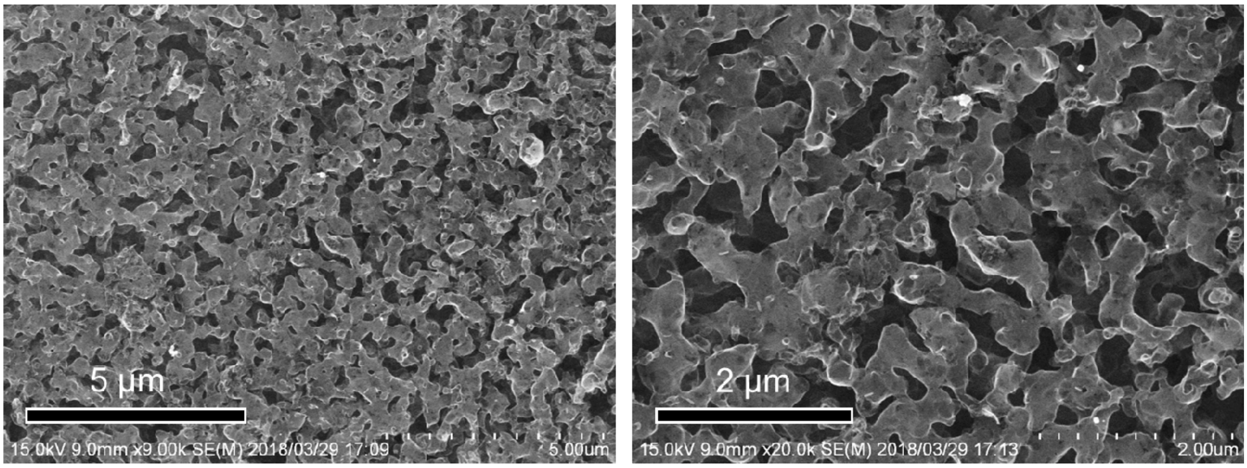


Figure S2. SEM images of NP-doped holey graphene.

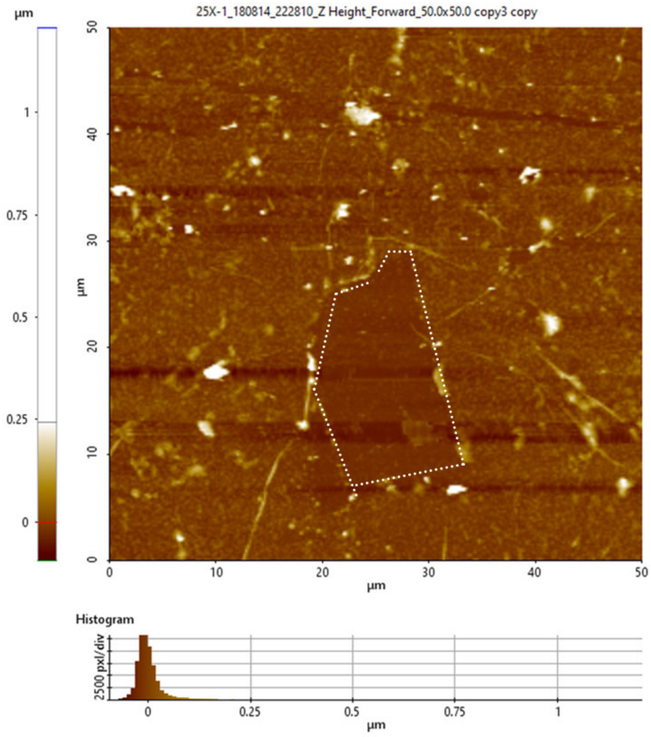


Figure S3. Typical AFM images of edge-rich holey graphene.

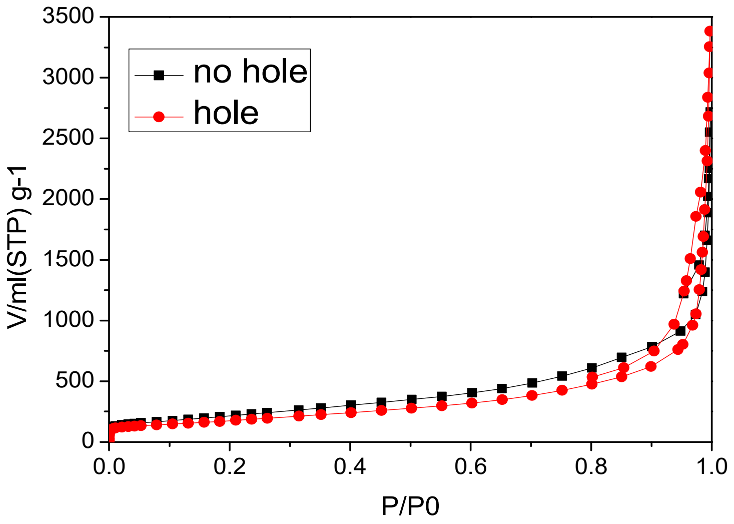


Figure S4. Nitrogen absorption and desorption measurements of NP-doped holey graphene and NP-doped graphene without holes. The NP-doped holey graphene contains small amount of residual $SiO_2$ nanoparticles so that the specific surface area becomes lower than that that of NP-doped graphene without holes.

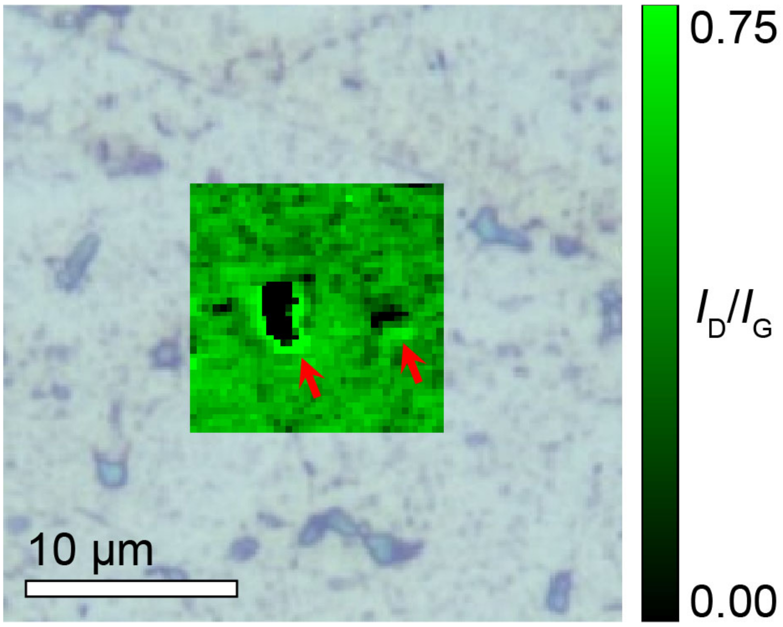


Figure S5 Raman mapping of NP-doped holey graphene for smaller hole size (2 μm and 500−600 nm) pointed by red allows.

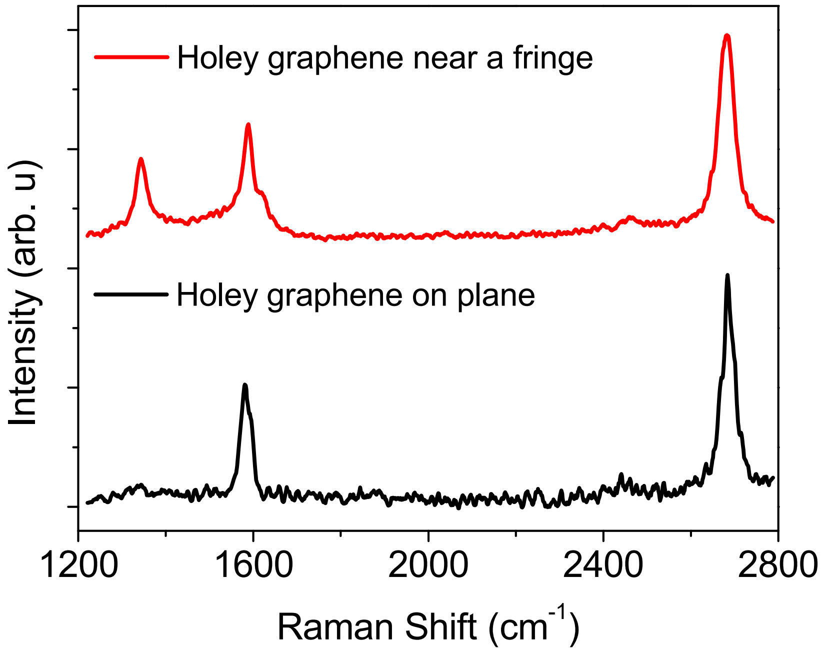


Figure S6 Raman spectra of holey graphene (non-doping) on plane region and fringe region.

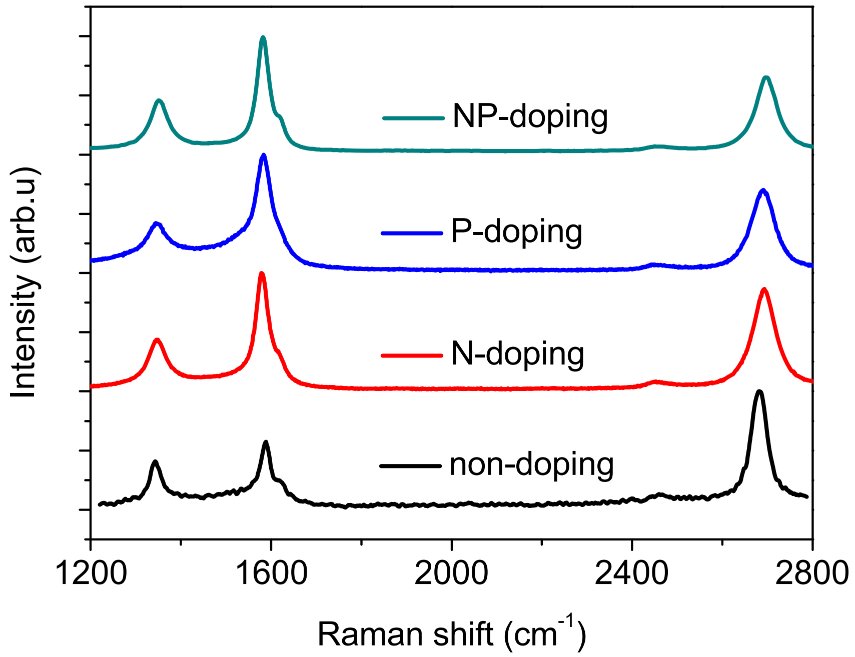


Figure S7. Raman spectra of chemically doped holey graphenes and holey graphene.

Table S1. Raman spectra measurements of the holey graphenes used in the main text. Unit of the spectra and line width is $cm^{-1}$.

| | D band/ Line width | G band Line width | D' band Line width | 2D band Line width | $I_D/I_G$ | $I_{2D}/I_G$ |
|---|---|---|---|---|---|---|
| holey graphene on plane | 1338 19 | 1582 11 | - | 2683 18 | 0.17 | 3.2 |
| holey graphene near a fringe | 1345 13 | 1585 13 | 1613 16 | 2682 20 | 0.72 | 3.1 |
| NP-doped holey graphene on plane | 1351 22 | 1581 11 | 1612 20 | 2697 26 | 0.46 | 1.56 |
| NP-doped holey graphene near a fringe | 1351 21 | 1581 12 | 1612 21 | 2697 27 | 0.91 | 1.64 |
| N-doped holey graphene near a fringe | 1347 21 | 1579 13 | 1611 19 | 2692 30 | 0.75 | 2.11 |
| P-doped holey graphene near a fringe | 1345 22 | 1581 14 | 1610 22 | 2690 30 | 0.80 | 1.77 |

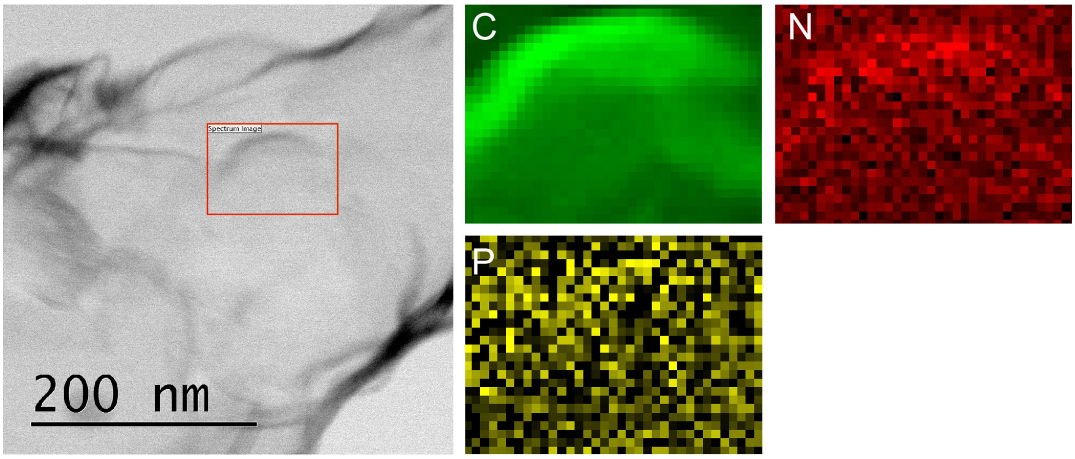


Figure S8. EELS mapping around wrinkle edge.

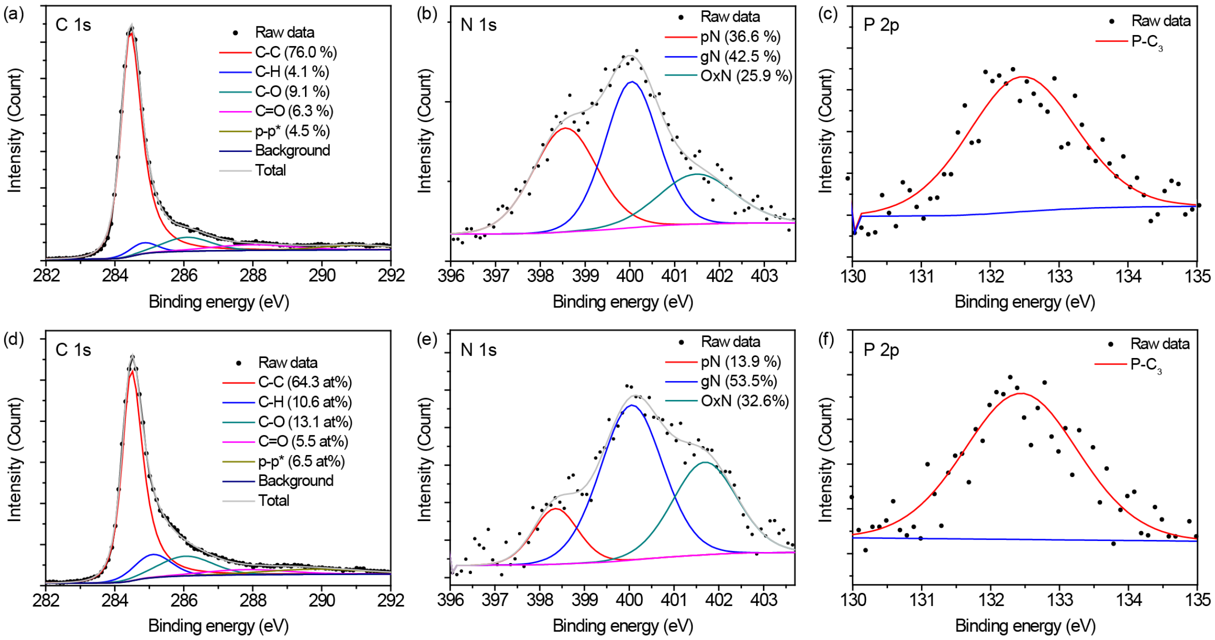


Figure S9. XPS spectra of C 1s, N 1s and P 1s for (a-c) NP-doped graphene with holes and (d-f) NP-doped graphene without holes. $Mo_2C$ in C 1s region were not detected for all chemically doped holey graphenes.

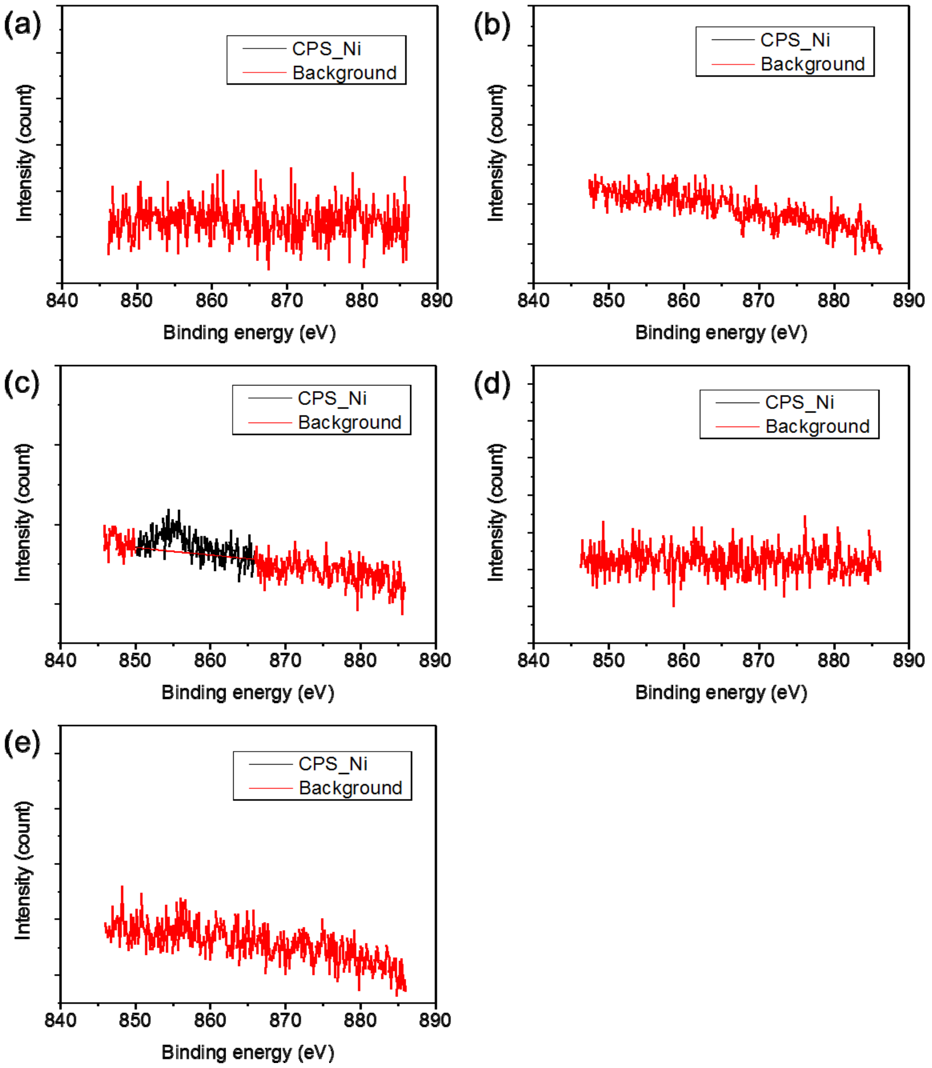

Figure S10. XPS spectra of Ni 2p for (a) holey graphene (<0.01 at%), (b) N-doped holey graphene (0.03 at%), (c) P-doped holey graphene (<0.01 at%), (d) NP-doped holey graphene (<0.01 at%) and (e) NP-doped graphene without holes (<0.01 at%).

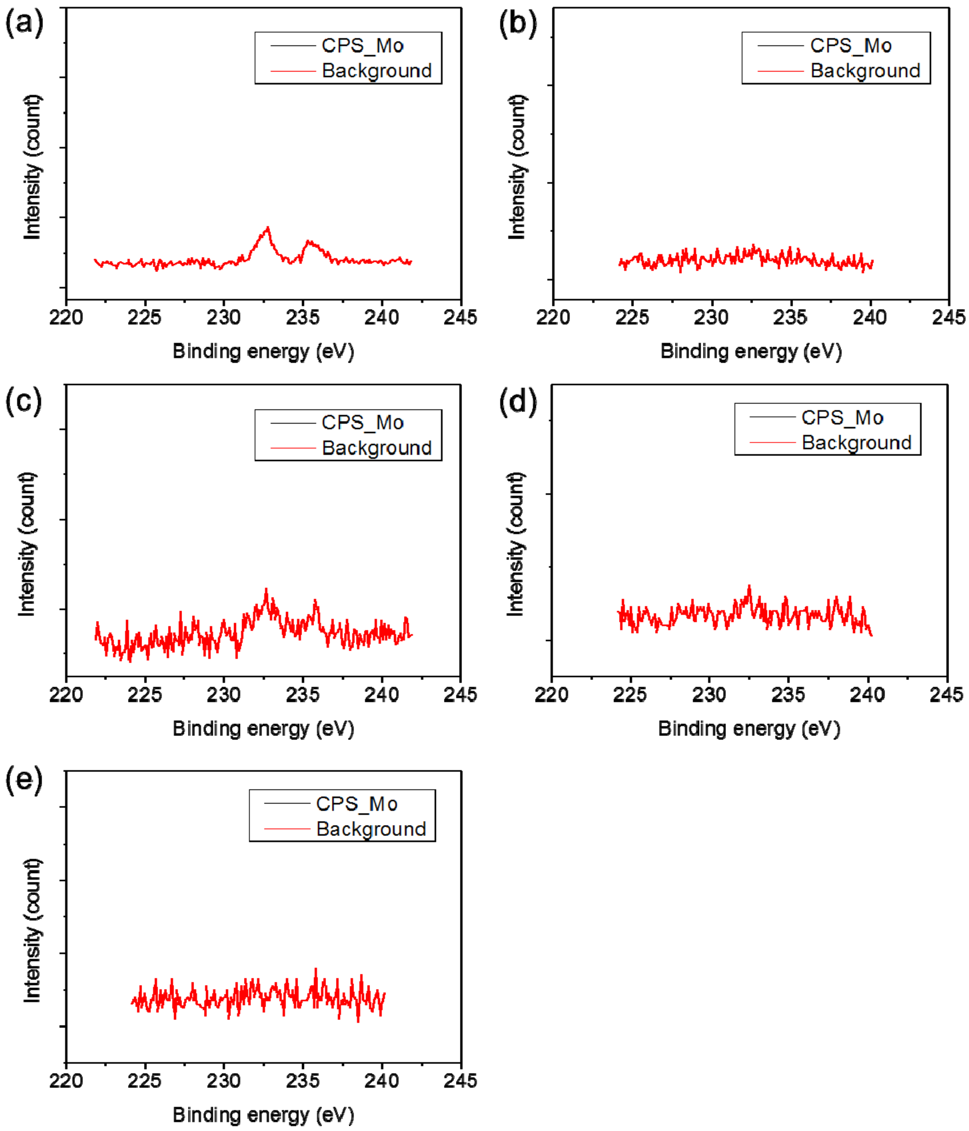


Figure S11. XPS spectra of Mo 3d for (a) holey graphene (0.03 at%), (b) N-doped holey graphene (<0.01 at%), (c) P-doped holey graphene (0.01 at%), (d) NP-doped holey graphene (<0.01 at%) and (e) NP-doped graphene without holes (<0.01 at%).

Table S2. Summary of XPS results of chemically doped holey graphenes.

| | Pyridinic N (at.%) | Graphitic N (at.%) | Oxidized N (at.%) | P-$C_3$ (at.%) | Oxidized P (at.%) | Total (at.%) |
|---|---|---|---|---|---|---|
| N-doped holey graphene | 1.16 | 0.62 | 0.23 | - | - | 2.01 |
| P-doped holey graphene | - | - | - | 0.12 | - | 0.12 |
| NP-doped holey graphene | 0.81 | 0.94 | 0.46 | 0.19 | - | 2.40 |
| NP-doped holey graphene after HER test | 0.15 | 0.84 | 0.55 | 0.12 | 0.05 | 1.71 |
| NP-doped graphene (non-hole) | 0.17 | 0.64 | 0.39 | 0.17 | - | 1.37 |

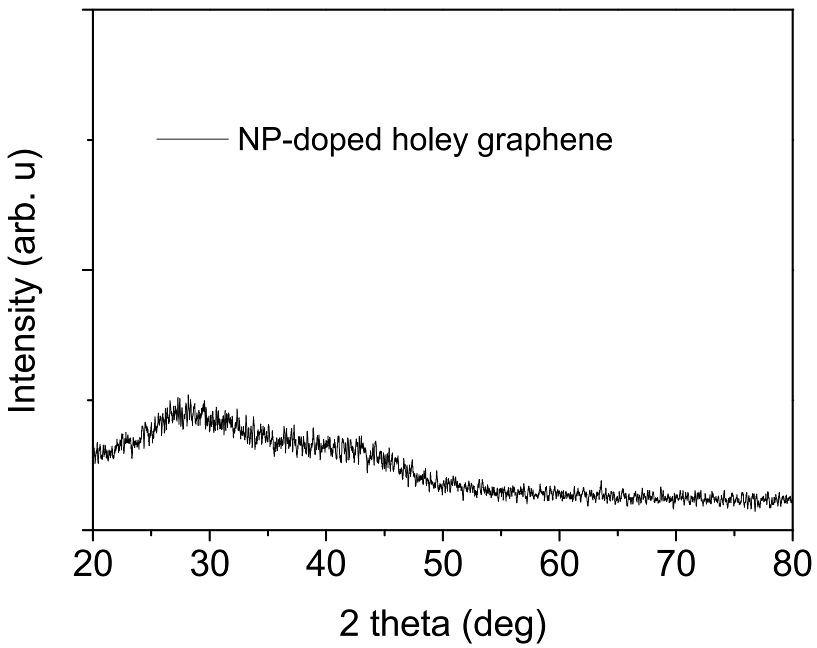


Figure S12. XRD spectra of NP-doped holey graphene.

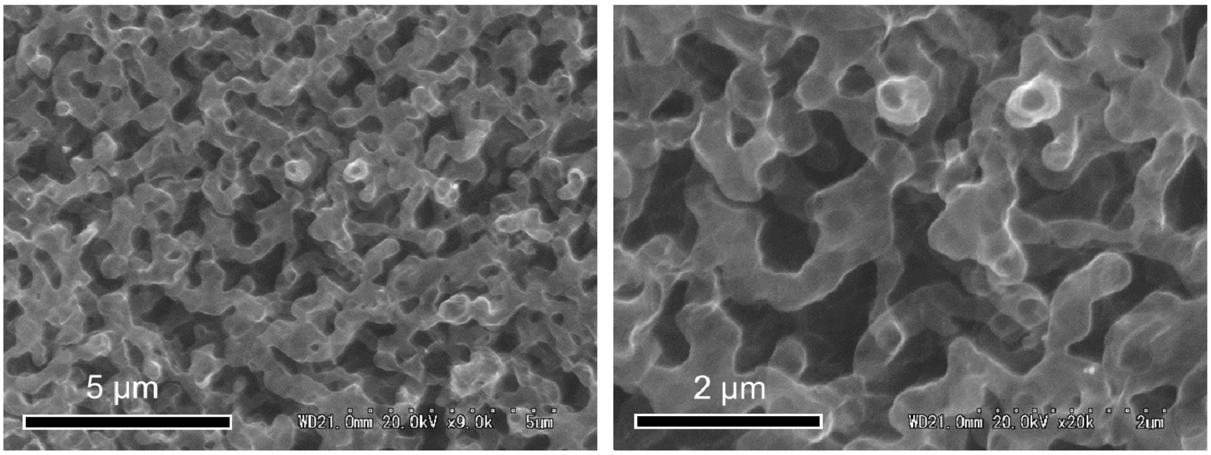


Figure S13. SEM images of NP-doped graphene without holes.

Table S3. Summary of TOF values of chemically doped holey graphene.

| Catalyst | Averaged TOF at electrode potential of 0.2 V (v.s. RHE) | TOF near the edge and plane at electrode potential of 0.2 V (v.s. RHE) |
|---|---|---|
| NP doped holey graphene | 0.64 | 468, 4.1 |
| NP doped graphene (non-hole) | 0.45 | N/A |
| N doped holey graphene | 0.24 | N/A |
| P doped holey graphene | 0.22 | 126, 3.8 |
| Non-doped graphene | 0.088 | 78, 0.96 |

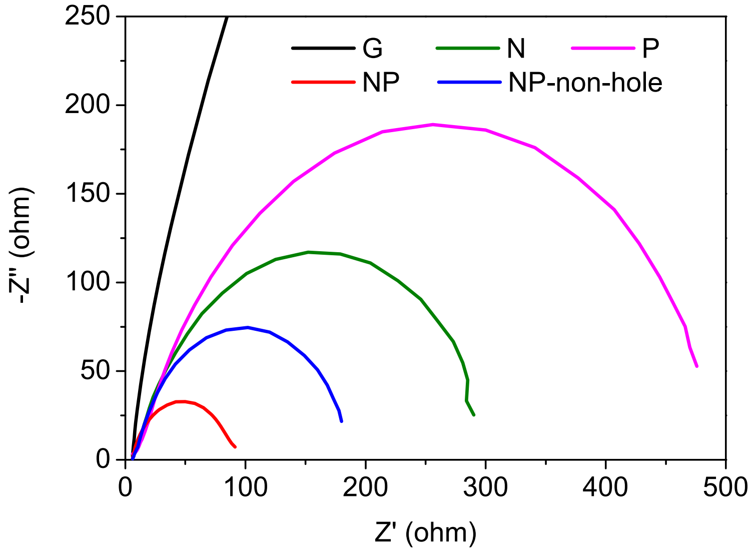


Figure S14. Electrical impedance of chemically doped holey graphenes at −200 mV (V v.s. RHE).

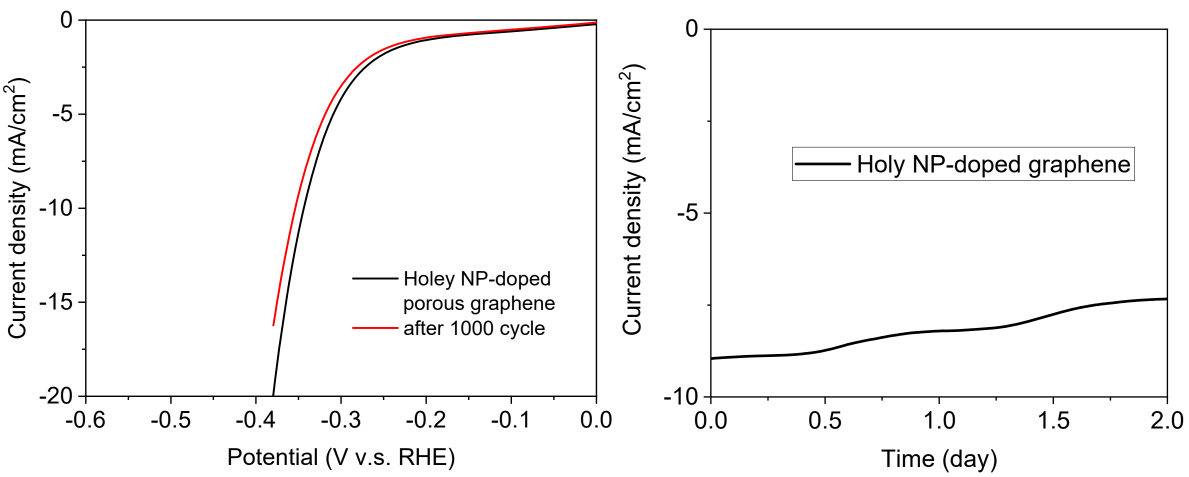


Figure S15. Cycling stability before and after 1000 cycling and durability test at the potential of −340 mV (V v.s. RHE) of NP-doped holey graphene.

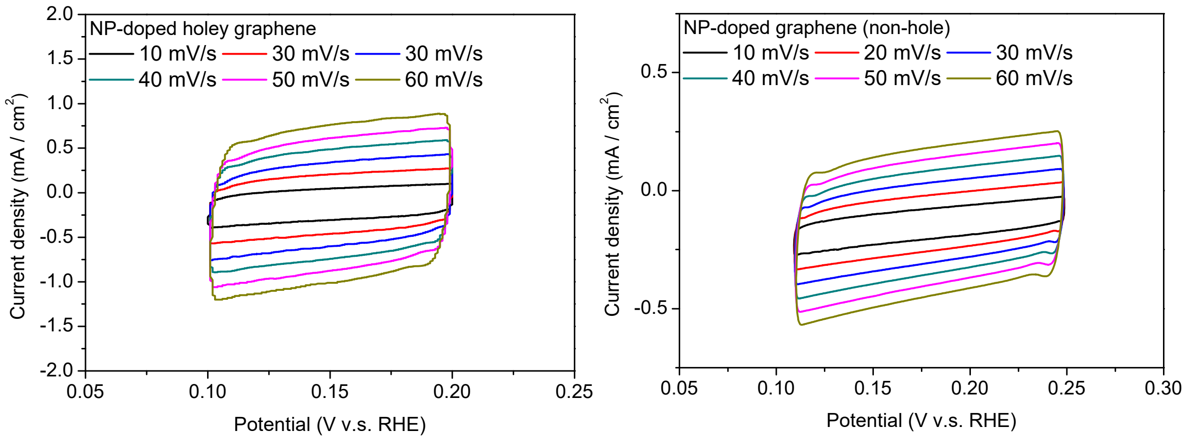


Figure S16. Typical cyclic voltammograms of NP-doped holey graphene in the double layer potential region and the differences in current density ($\Delta J = J_a - J_c$) at 150 mV and 175 mV (V v.s. RHE) plotted against scan rate for the estimation of double layer capacitance ($C_{dl}$) in 0.5 M aqueous $H_2SO_4$ electrolyte.

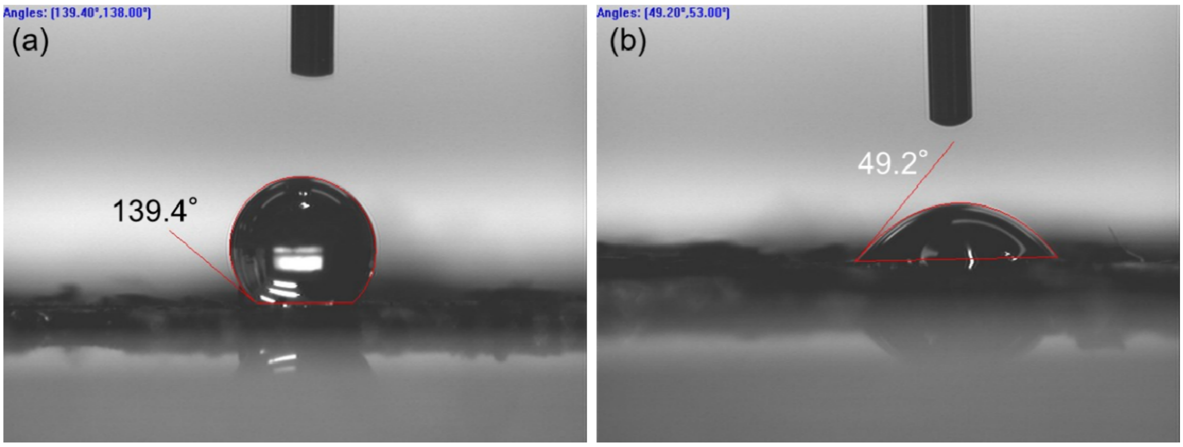


Figure S17. Typical contact angle of (a) NP-doped graphene without holes and (b) NP-doped graphene with holes.

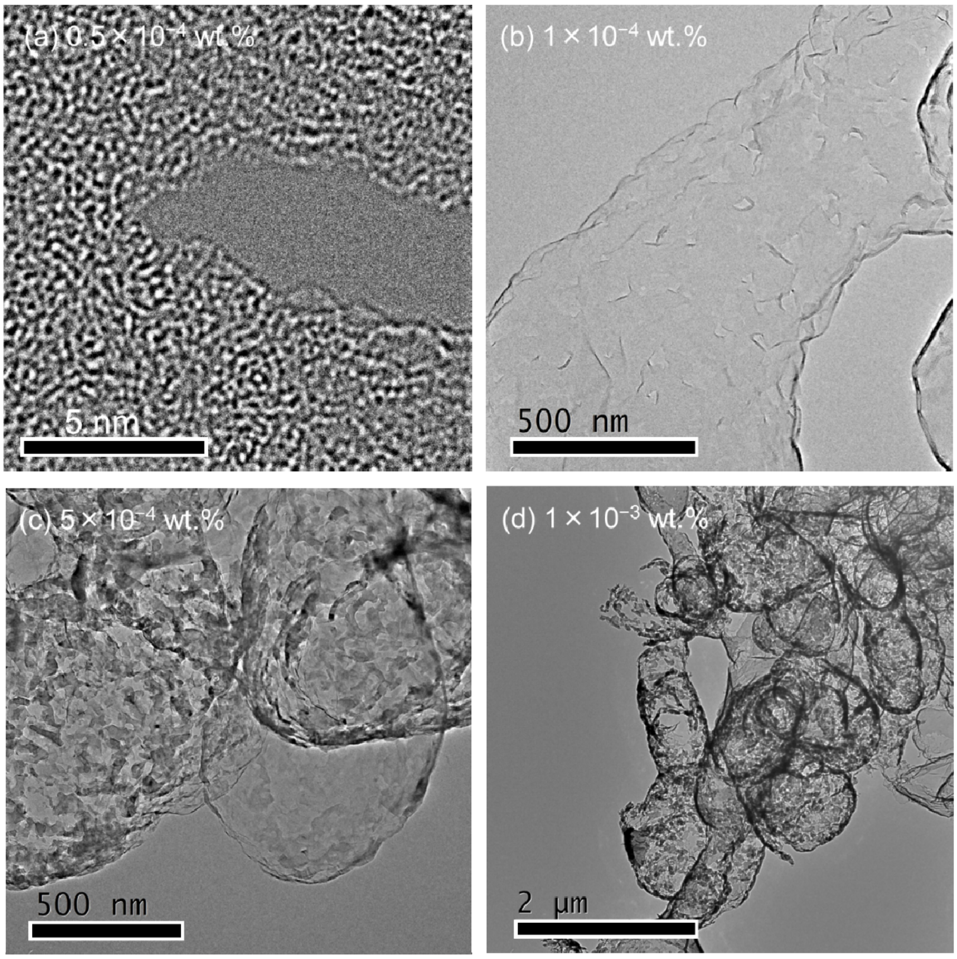


Figure S18. TEM images of different amount of $SiO_2$ nanoparticles on NP-doped holey graphene. (a) $0.5\times10^{-4}$ wt.% samples, (b) $1\times10^{-4}$ wt.% samples, (c) $5\times10^{-4}$ wt.% samples and (d) $1\times10^{-3}$ wt.% samples showed hole structures. The $1\times10^{-3}$ wt.% samples cannot keep their structures due to many holes (very fragile). The fringe region on the all samples also show disordered graphene lattice.

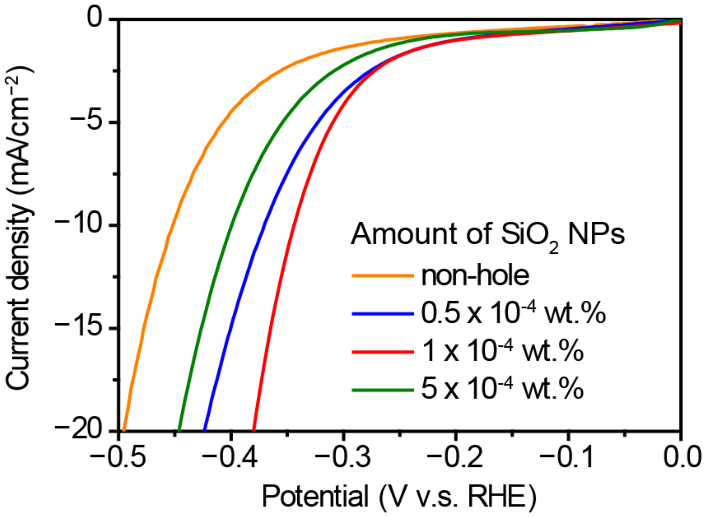


Figure S19. $SiO_2$ level dependence of hydrodynamic voltammograms of graphene samples in 0.5 M aqueous $H_2SO_4$ electrolyte, loaded at 3.0 mg/cm$^2$.

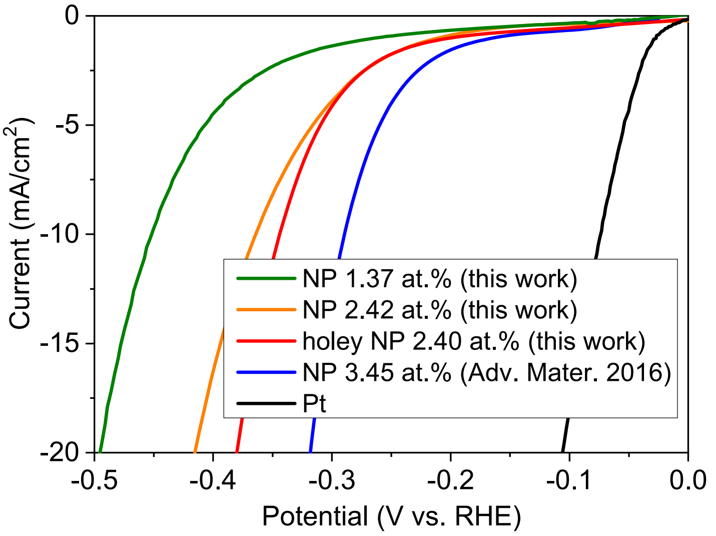


Figure S20. Chemical dopant concentration dependence of HER performances on NP-doped graphene in 0.5 M $H_2SO_4$ electrolyte. The 3.45 at.% sample was reproduced from *Adv. Mater.* **2016**, *28,* 10644–10651.

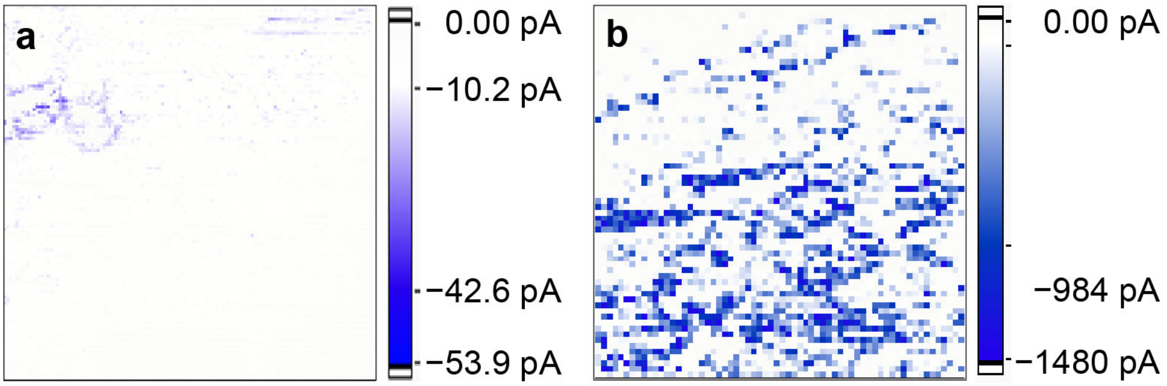


Figure S21. HER current mapping of (a) P-doped holey graphene and (b) N-doped holey graphene. The N-doped sample failed to form water meniscus and survey overall current due to the hydrophilic characters of N-doped graphene.

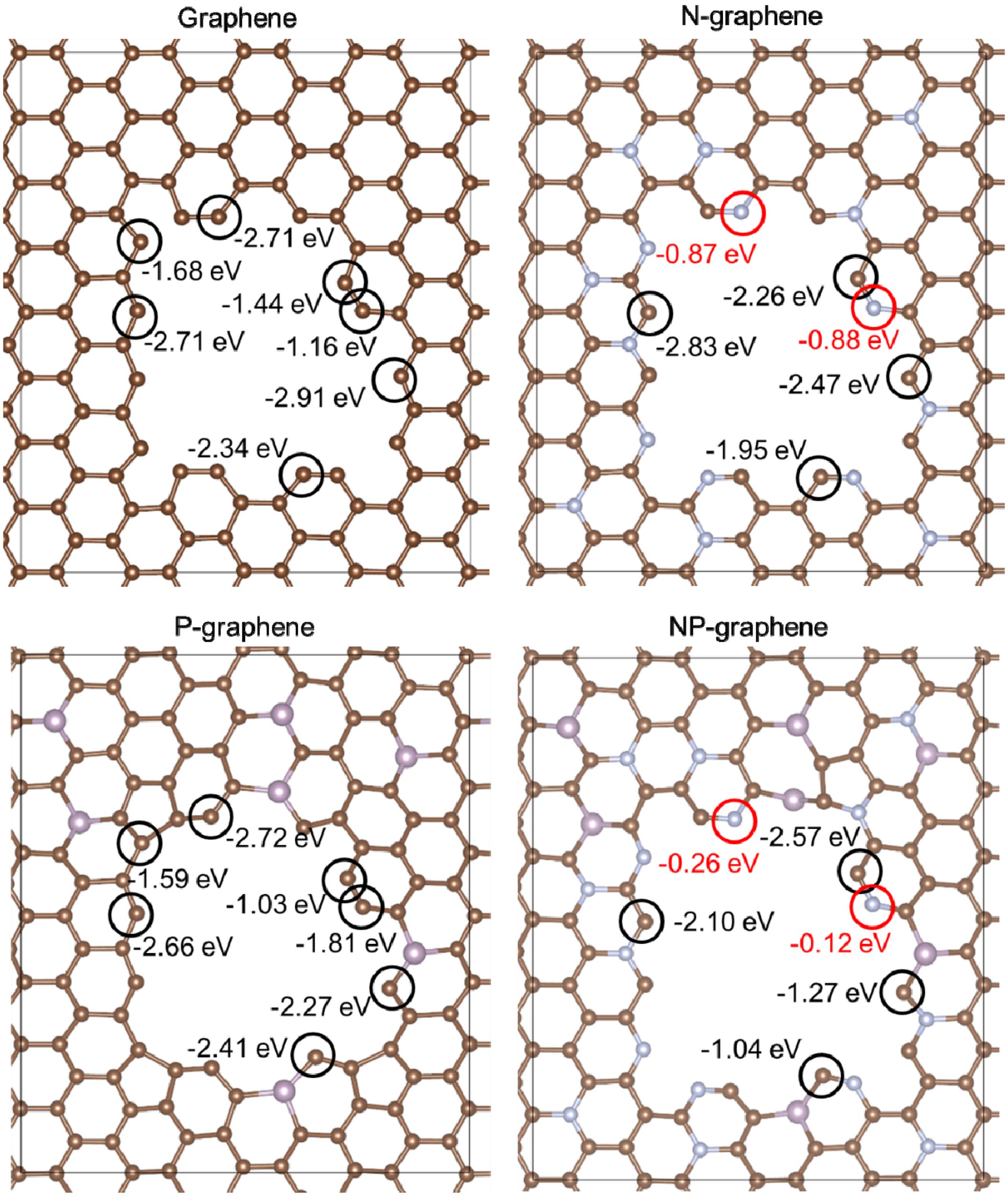


Figure S22. DFT-calculated $\Delta G_{H^*}$ values at individual sites. Only one H atom was adsorbed on each site.

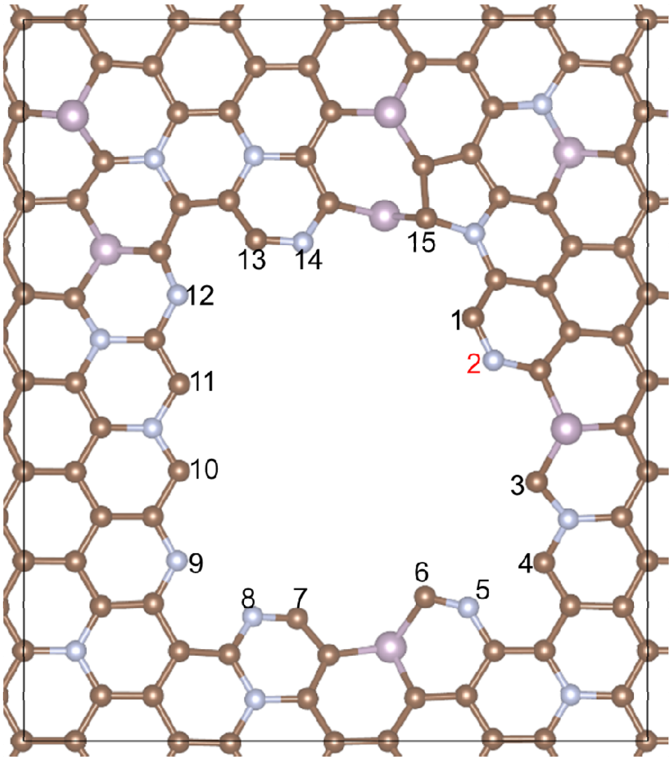


| Site | ΔG$_H$ (eV) Graphene | NP-graphene |
|---|---|---|
| 1 | -1.44 | -2.57 |
| 2 | -1.16 | -0.12 |
| 3 | -2.91 | -1.27 |
| 4 | -2.41 | -1.68 |
| 5 | - | - |
| 6 | -2.35 | -1.05 |
| 7 | -2.88 | -1.98 |
| 8 | -1.71 | -0.74 |
| 9 | -1.71 | -0.67 |
| 10 | -2.72 | -2.20 |
| 11 | -2.71 | -2.10 |
| 12 | -1.68 | -2.18 |
| 13 | -1.68 | -1.94 |
| 14 | -2.72 | -0.26 |
| 15 | -2.74 | -1.06 |

Figure S23. Structure of NP-graphene and DFT-calculated $\Delta G_{H*}$ values at all the individual sites. Only one H atom was adsorbed on each site. The most active sites are indicated by red.

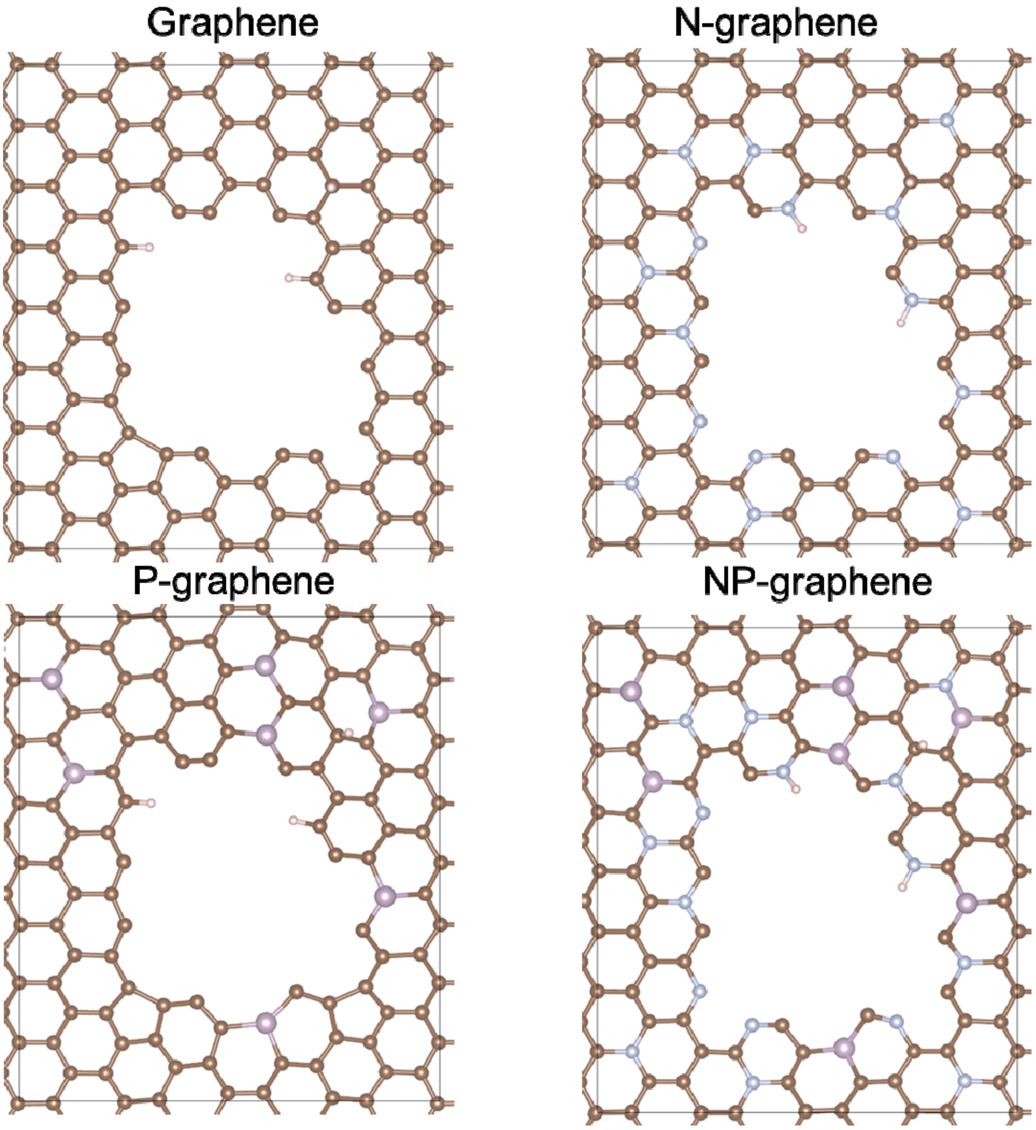


Figure S24. DFT-calculation model of holey graphene ($\Delta G_{H*}$=−0.61 eV), N-doped holey graphene ($\Delta G_{H*}$=−0.46 eV), P-doped holey graphene ($\Delta G_{H*}$=−0.62 eV) and NP-doped holey graphene ($\Delta G_{H*}$=−0.02 eV) with a single hole. Blue, purple, and white balls represent nitrogen, phosphorus, and hydrogen atoms, respectively. Nitrogen can take two major configurations of pyridinic nitrogen (secondary form) and graphitic nitrogen (tertiary form) and phosphorus can take only one major configuration (tertiary form) as illustrated in the model.

Table S4. Calculated $\Delta G_{H*}$ values with different exchange-correlation functionals.

| | PBE | RPBE |
|---|---|---|
| Holey graphene | −0.61 eV | −0.57 eV |
| N-doped holey graphene | −0.46 eV | −0.41 eV |
| P-doped holey graphene | −0.62 eV | −0.58 eV |
| NP-doped holey graphene | −0.02 eV | −0.03 eV |

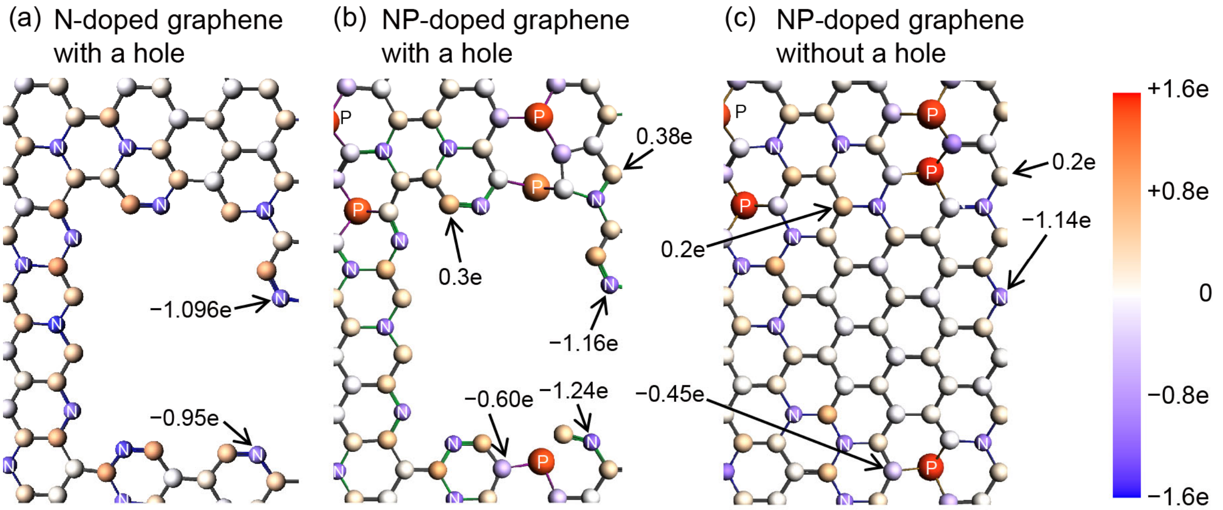


Figure S25. Charge distribution mapping of N-doped and NP-doped graphene with and without a nanohole, and a comparison of charges on carbon atoms. N and P atoms have strong positive and negative charges, respectively. P dopants enhance further charge accommodation of N atoms. The scale bar displays the charge intensity.

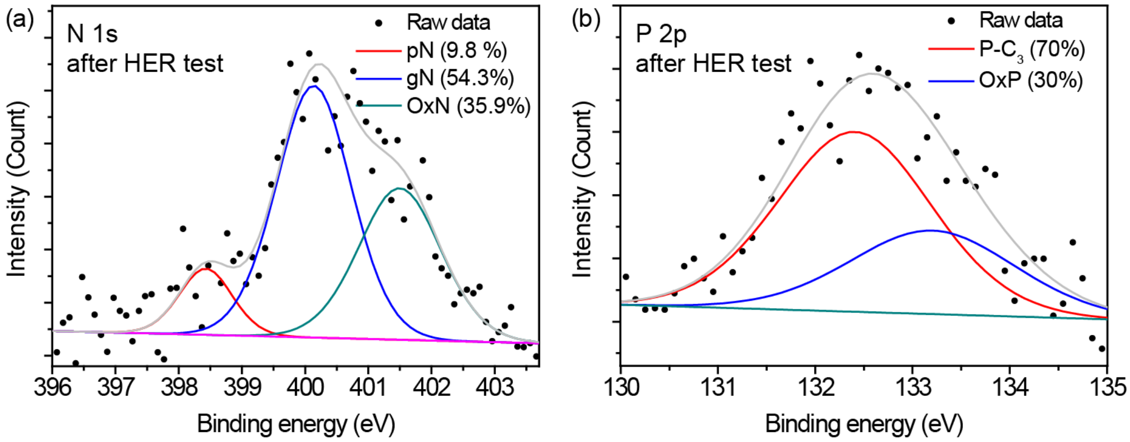


Figure S26. XPS spectra of (a) N1s and (b) P2p on the NP-doped holey graphene sample after the HER test.

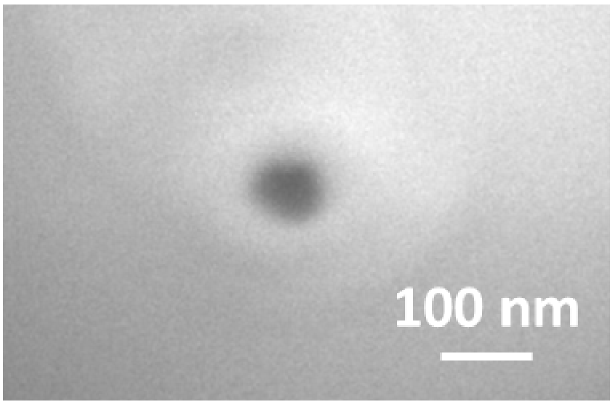


Figure S27. Typical SEM image of nanopipette used for HER current mapping. Black spot demonstrated the diameter of nanopipette hole.

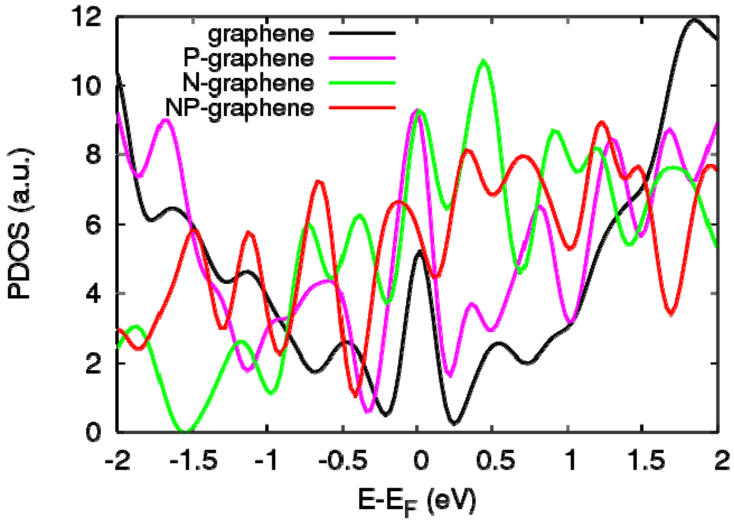


Figure S28. The projected density of states of the C atoms. The large state exists at the Fermi level.

Table S5. Onset overpotentials, Tafel slopes, exchange current density and operating potential at 10 mA/cm$^2$ of metal-free carbon/graphene materials reported in the literatures.

| Catalyst | Structure | Onset over-potentials (mV) | Tafel slope (mV/dec) | Exchange current density (μA/cm$^2$) | η(mV)@*j*= 10 mA/cm$^2$ | Reference |
|---|---|---|---|---|---|---|
| Holey graphene | 3D structure | 405 | 171 | 4.8 | 571 | This work |
| N-doped holey graphene | 3D structure | 303 | 164 | 13.2 | 477 | This work |
| P-doped holey graphene | 3D structure | 350 | 154 | 4.1 | 522 | This work |
| NP-doped holey graphene | 3D structure | 223 | 118 | 12.4 | 344 | This work |
| NP-doped graphene (non-holes) | 3D structure | 280 | 145 | 7.7 | 453 | This work |
| graphite | powder | - | 206 | - | - | ACS Nano, 8, 5290 (2014). |
| N-doped graphene | 2D sheets | 330 | 116 | $7.0 \times 10^{-2}$ | 490 | ACS Nano, 8, 5290 (2014). |
| P-doped grpahene | 2D sheets | 370 | 133 | $9.0 \times 10^{-3}$ | 553 | ACS Nano, 8, 5290 (2014). |
| N,P-doped graphene | 2D sheets | 290 | 91 | $2.4 \times 10^{-1}$ | 420 | ACS Nano, 8, 5290 (2014). |
| N-doped graphene | 3D structure | 242 | 129 | 2.14 | 484 | ChemCatChem 7, 3873 (2015). |
| P-doped graphene | 3D structure | 105 | 118 | 2.61 | 409 | ChemCatChem 7, 3873 (2015). |
| porous graphene 800°C | 3D structure | 510 | 270 | 0.37 | - | Angew. Chem. Int. Ed., 54, 2131 (2015) |
| N-doped porous graphene 800°C | 3D structure | 400 | 232 | 3.7 | 559 | Angew. Chem. Int. Ed., 54, 2131 (2015) |

Table S6. Onset overpotentials, Tafel slopes, exchange current density and operating potential at 10 mA/cm$^2$ of metal-free carbon/graphene materials reported in the literatures.

| Catalyst | Structure | Onset over-potentials (mV) | Tafel slope (mV/dec) | Exchange current density (μA/cm$^2$) | η(mV)@*j*= 10 mA/cm$^2$ | Reference |
|---|---|---|---|---|---|---|
| porous graphene 750°C | 3D structure | 450 | 139 | 0.2 | 650 | *Adv. Mater.,* 28, 10644 (2016) |
| N-doped porous graphene 750°C | 3D structure | 330 | 163 | 4.0 | 550 | *Adv. Mater.,* 28, 10644 (2016) |
| P-doped porous graphene 750°C | 3D structure | 230 | 204 | 65 | 430 | *Adv. Mater.,* 28, 10644 (2016) |
| NP-doped porous graphene 750°C | 3D structure | 160 | 102 | 24 | 290 | *Adv. Mater.,* 28, 10644 (2016) |
| N,P-doped carbon | 3D structure | 60 | 89 | - | - | *Angew. Chem. Int. Ed.,* 55, 2230 (2016). |
| N-doped carbon | 2D sheets | - | 159.3 | - | 620 | *ACS Nano,* 11, 7, 7293 (2017). |
| N,P-doped carbon | 2D sheets | - | 118.2 | - | 550 | *ACS Nano,* 11, 7, 7293 (2017). |
| 1T-$MoS_2$ sheets | 2D sheets | - | 43 | - | 187 | *J. Am. Chem. Soc.,* 135, 10274 (2013). |
| mesoporous $MoS_2$ | 3D structure | 150-200 | 50 | 0.69 | - | Nat. Mater., 11, 963 (2012). |
| defect-rich $MoS_2$ sheets | 2D sheets | 120 | 50 | 8.91 | - | Adv. Mater., 25, 5807 (2013). |
| monolayer $MoS_2$ film | 2D thick film | 200 | 41 | 0.63 | - | Energy Environ. Sci., 5, 6136 (2012). |
| monolayer $MoS_2$ film | 2D thick film | - | 140-150 | 0.16 | - | Nano Lett., 14, 553 (2014). |
| monolayer $MoS_2$ on porous gold | 3D structure | 115 | 46 | 0.419 | 226 | Adv. Mater., 26, 8023 (2014). |

**References**

[S1] Jothi, P. R.; Kannan, S.; Velayutham, G. Enhanced methanol electro-oxidation over in-situ carbon and graphene supported one dimensional $NiMoO_4$ nanorods. *J. Pow. Sour.* **2015**, *277*, 350−359.
[S2] Fang, M.; Gao, W.; Dong, G.; Xia, Z.; Yip, S.; Qin, Y.; Qu, Y.; Ho, J. C. Hierarchical NiMo-based 3D electrocatalysts for highly-efficient hydrogen evolution in alkaline conditions. *Nano Energy* **2016**, *27*, 247–254.
[S3] Hu, K.; Jeong, S.; Wakisaka, M.; Fujita, J.; Ito, Y. Bottom-Up Synthesis of Porous NiMo Alloy for Hydrogen Evolution Reaction, *Metals*, **2018**, *8*, 83.
[S4] Burke, M. S.; Enman, L. J.; Batchellor, A. S.; Zou, S.; Boettcher, S. W. Oxygen Evolution Reaction Electrocatalysis on Transition Metal Oxides and (Oxy)hydroxides: Activity Trends and Design Principles. *Chem. Mater.* **2017**, *29*, 120−140.
[S5] Luo, J.; Im, J.-H.; Mayer, M. T.; Schreier, M.; Nazeeruddin, M. K.; Park, N.-K.; Tilley, S. D.; Fan, H. J.; Grätzel, M. Water photolysis at 12.3% efficiency via perovskite photovoltaics and Earth-abundant catalysts. *Science* **2014**, *345*, 1593−1596.
[S6] Suntivich, J.; May, K. J.; Gasteiger, H. A.; Goodenough, J. B.; Shao-Horn, Y. A perovskite oxide optimized for oxygen evolution catalysis from molecular orbital principles. *Science* **2011**, *334*, 1383−1385.
[S7] McCrory, C. C. L.; Jung, S.; Ferrer, I. M.; Chatman, S. M.; Peters, J. C.; Jaramillo, T. F. Benchmarking Hydrogen Evolving Reaction and Oxygen Evolving Reaction Electrocatalysts for Solar Water Splitting Devices. *J. Am. Chem. Soc*. **2015**, *137*, 4347−4357.
[S8] Long, X.; Li, G.; Wang, Z.; Zhu, H.; Zhang, T.; Xiao, S.; Guo, W.; Yang, S. Metallic Iron-Nickel Sulfide Ultrathin Nanosheets as a Highly Active Electrocatalyst for Hydrogen Evolution Reaction in Acidic Media. *J. Am. Chem. Soc.* **2015**, *137*, 11900−11903.
[S9] Wang, T. ; Guo, Y. ; Zhou, Z. ; Chang, X. ; Zheng, J.; Li, X. Ni–Mo Nanocatalysts on N-Doped Graphite Nanotubes for Highly Efficient Electrochemical Hydrogen Evolution in Acid *ACS Nano* **2016**, *10*, 10397–10403.
[S10] Lukowski, M. A.; Daniel, A. S.; Meng, F.; Forticaux, A.; Li, L.; Jin, S. Enhanced Hydrogen Evolution Catalysis from Chemically Exfoliated Metallic $MoS_2$ Nanosheets *J. Am. Chem. Soc.* **2013**, *135*, 10274−10277.
[S11] Long, X.; Li, G.; Wang, Z.; Zhu, H.; Zhang, T.; Xiao, S.; Guo, W.; Yang, S. Metallic Iron–Nickel Sulfide Ultrathin Nanosheets As a Highly Active Electrocatalyst for Hydrogen Evolution Reaction in Acidic Media. *J. Am. Chem. Soc.* **2015**, *137*, 11900−11903.
[S12] Benck, J. D.; Chen, Z. B.; Kuritzky, L. Y.; Forman, A. J.; Jaramillo, T. F. Amorphous Molybdenum Sulfide Catalysts for Electrochemical Hydrogen Production: Insights into the Origin of their Catalytic Activity. *ACS Catal.* **2012**, *2*, 1916–1923.
[S13] Chen, Z.; Cummins, D.; Reinecke, B. N.; Clark, E.; Sunkara, M. K.; Jaramillo, T. F. Core–shell $MoO_3$–$MoS_2$ Nanowires for Hydrogen Evolution: A Functional Design for Electrocatalytic Materials. *Nano Lett.* **2011**, *11*, 4168–4175.
[S14] Seokhee Shin, Zhenyu Jin, Do Hyun Kwon, Ranjith Bose, and Yo-Sep Min, *Langmuir*, **31**, 1196−1202 (2015).
[S15] G. Kresse, J. Hafner, *J. Phys. Condens. Matter* **1994**, *6,* 8245–8257.
[S16] P. E. Blöchl, *Phys. Rev. B* **1994**, *50,* 17953–17979.
[S17] J. P. Perdew, K. Burke, M. *Phys. Rev. Lett.* **1996**, *77*, 3865–3868.
[S18] S. Grimme, J. Antony, S. Ehrlich, H. Krieg, *J. Chem. Phys.* **2010**, *132*, 154104.
[S19] S. Grimme, S. Ehrlich, L. Goerigk, *J. Comput. Chem.* **2011**, *32,* 1456–1465.
[S20] Ito, Y.; Shen, Y.: Hojo, D.; Itagaki, Y.; Fujita, T.; Chen, L.; Aida, T. Tang, Z.; Adschiri, T.; Chen, M. W. Correlation between Chemical Dopants and Topological Defects in Catalytically Active Nanoporous Graphene. Adv. Mater. 2016, 28, 10644–10651.

[S21] Hammer, B.; Hansen, L. B.; J. K. Norskov. Improved adsorption energies within density-functional theory using Perdew-Burke-Ernzerhof functionals, Phys. Rev. B **1999**, 59, 7413-7421.
[S22] Zhao, M.; Anderson A. B. Theory of Hydrogen Deposition and Evolution on Cu(111) Electrodes, Journal of The Electrochemical Society, **2017**, 164 H691-H695.
[S23] Yao Zheng, Yan Jiao, Yihan Zhu, Lu Hua Li, Yu Han, Ying Chen, Aijun Du, Mietek Jaroniec & Shi Zhang Qiao, Hydrogen evolution by a metal-free electrocatalyst, *Nature Communications* **5**,3783 (2014).
[S24] Y. Takahashi, A. Kumatani, H. Munakata, H. Inomata, K. Ito, K. Ino, H. Shiku, P. R. Unwin, Y. E. Korchev, K. Kanamura & T. Matsue. Nanoscale visualization of redox activity at lithium-ion battery cathodes, Nat Commun 5, 5450 (2014).
